# MicroRNA miR-328 Regulates Zonation Morphogenesis by Targeting CD44 Expression

**DOI:** 10.1371/journal.pone.0002420

**Published:** 2008-06-18

**Authors:** Chia-Hui Wang, Daniel Y. Lee, Zhaoqun Deng, Zina Jeyapalan, Shao-Chen Lee, Shireen Kahai, Wei-Yang Lu, Yaou Zhang, Burton B. Yang

**Affiliations:** 1 Sunnybrook Research Institute, Sunnybrook Health Sciences Centre, Toronto, Canada; 2 Department of Laboratory Medicine and Pathobiology, University of Toronto, Toronto, Canada; 3 Department of Anaesthesia, University of Toronto, Toronto, Canada; 4 Life Science Division, Graduate School at Shenzhen, Tsinghua University, Shenzhen, China; Wellcome Trust Sanger Institute, United Kingdom

## Abstract

Morphogenesis is crucial to initiate physiological development and tumor invasion. Here we show that a microRNA controls zonation morphogenesis by targeting hyaluronan receptor CD44. We have developed a novel system to study microRNA functions by generating constructs expressing pre-miRNAs and mature miRNAs. Using this system, we have demonstrated that expression of *miR-328* reduced cell adhesion, aggregation, and migration, and regulated formation of capillary structure. Protein analysis indicated that *miR-328* repressed CD44 expression. Activities of luciferase constructs harboring the target site in CD44, but not the one containing mutation, were repressed by *miR-328*. Zonation morphogenesis appeared in cells transfected by miR-328: miR-328-transfected cells were present on the surface of zonating structures while the control cells stayed in the middle. *MiR-328*-mediated CD44 actions was validated by anti-CD44 antibody, hyaluronidase, CD44 siRNA, and CD44 expression constructs. *In vivo* experiments showed that CD44-silencing cells appeared as layers on the surfaces of nodules or zonating structures. Immuno-histochemistry also exhibited CD44-negative cells on the surface layers of normal rat livers and the internal zones of Portal veins. Our results demonstrate that *miR-328* targets CD44, which is essential in regulating zonation morphogenesis: silencing of CD44 expression is essential in sealing the zonation structures to facilitate their extension and to inhibit complex expansion.

## Introduction

Once ignored completely or overlooked as cellular detritus, small RNA fragments (microRNA or miRNA) that were discovered over a decade ago recently took many by surprise because of their widespread expression and functions [1], [2], [3]. miRNAs are single-stranded RNA of 18-24 nucleotides, which are generated by sequential processing of long RNA transcripts by two key RNase III proteins, Drosha and Dicer [4], [5], [6], [7], [8]. miRNA functions as a guide molecule in post-transcriptional gene silencing by partially pairing with the 3′-untranslated region (UTR) of target mRNAs, leading to translational repression [9]. miRNAs have key roles in diverse regulatory pathways, including control of development [10], [11], [12], [13], cell proliferation [14], [15], [16], differentiation [17], [18], [19], [20], [21], apoptosis [22], [23], cell cycle progression [2], [24], [25], immuno-response [21], [26], [27], protein secretion [28], [29], viral infection [30], [31], [32], [33], [34], [35], tumorigenesis [36], [37], [38], [39], [40] and many other physiological or pathological processes[21], [41], [42], Recent studies indicated that microRNAs are important for tissue morphogenesis [43], [44], [45], [46]. However, it is not known which microRNA(s) is the key player in this process.

In animals, miRNA genes are transcribed to generate long primary transcripts (called pri-miRNAs), which are first cropped by the RNase III-type enzyme Drosha to generate some hairpin intermediates (called pre-miRNAs) in the nucleus [8], [47]. Pre-miRNAs are then exported to the cytoplasm by exportin-5, a member of the Ran-dependent nuclear transport receptor family [48]. Following arrival in the cytoplasm, pre-miRNAs are subjected to another processing step by Dicer, a cytoplasmic RNase III-type enzyme [49], [50]. Although miRNAs have emerged as key regulators of gene expression, our understanding of the specific roles of miRNAs has been limited due to the difficulty in tracking the functions of a particular miRNA. Exogenous miRNAs are readily degraded by enzymes, making it impossible to obtain stable cell lines expressing miRNAs for long-term studies *in vitro* or *in vivo*. Although expression of a large fragment of DNA harboring the promoter and sequence for microRNA(s) has made stable expression possible [51], in many cases microRNAs are expressed as a cluster, making it difficult to distinguish the function of a particular microRNA. To allow long-term studies of miRNA functioning *in vitro* and *in vivo*, we have developed an expression vector harboring two copies of pre-microRNAs, a GFP tracking unit, and an antibiotic selection marker. This allows stable expression of high levels of the miRNA of interest in cells for functional and complementation studies. Using this technique, we have successfully expressed a number of miRNAs in mammalian cells including *miR-328*. Using the software available [52], [53], [54], we have found that *miR-328* potentially targets a number of cell adhesion molecules including CD44. This study was designed to investigate the role of *miR-328* in affecting cell activities by targeting CD44 expression.

## Results and Discussion

### Cell Activities Affected by *miR-328*

**Figure 1:**
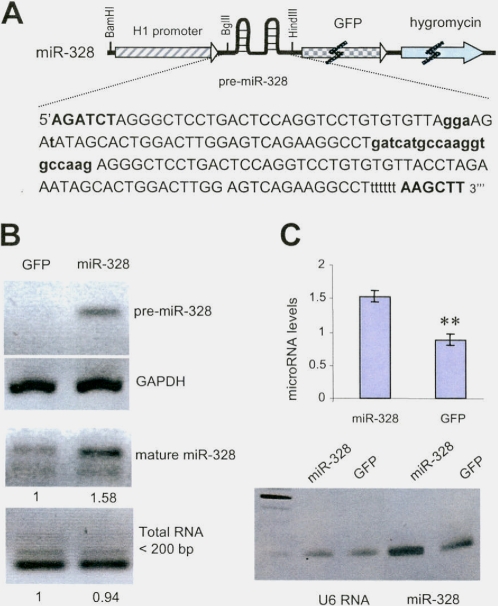
Construction and expression of *miR-328.* (A) Diagram of a construct expressing GFP, a neomycin-resistance gene, and two hairpin structures of pre-miR-328. The bolded and capitalized letters represent two restriction sites BglII and HindIII. The bolded lower case sequence represents an artifact sequence inserted between two pre-miRNAs. Six “t”s were added to terminate transcription. A few lower case letters represent changes of nucleotides that are not part of the mature *miR-328* but are useful for PCR purpose. (B) RT-PCR of pre-miR-328 using RNA prepared from A431 cells stably transfected with miR-328 and a control vector, using two primers miR-328N and miR-328C, confirming expression of pre-miR-328. As a control, Gapdh was amplified with two primers huGapdh131F and huGapdh380R. RT-PCR of mature *miR-328* using RNA prepared from A431 cells stably transfected with miR-328 and a control vector, confirming proper processing of *miR-328*. (C) Real-time PCR analysis confirmed expression of mature *miR-328*. **, p<0.01. Error bars, SD (n = 3). The end products of real-time PCR were subjected to agarose gel electrophoresis (*lower*).

To investigate the role of a microRNA in morphogenesis, it is essential to maintain its function for a period of time. We have generated a construct that can express the pre-microRNA of *miR-328* and GFP, with an antibiotic selection marker (Figure 1A, all primers used in this study are listed as Supplementary Table S1). The advantage of this construct is the stability of miRNA expression after stable transfection. Furthermore, transfection of this construct allows selection with neomycin, as well as rescue of positive clones by monitoring GFP-positive colonies, or by sorting GFP-positive cells. We have detected the expression of pre-miR-328 by RT-PCR using RNA prepared from A431 cells stably transfected with miR-328 (Figure 1B). The pre-miR-328 was then successfully processed to mature *miR-328*, as detected by RT-PCR with primers designed by us (Figure 1B, for diagram illustrating reaction see Supplementary Figure S1A and Table S1). The advantage of using RT-PCR for mature microRNA analysis was the limited primer used for annealing to template to avoid non-specific detection. In the reaction, we only designed 15-base-pair-annealing for reverse transcription and 12-base-pair-annealing for PCR. This should greatly reduce non-specific interaction that can occur during Northern hybridization. To assure that processing of the expressed *miR-328* did not interfere with the RNAi/miRNA pathways, we analyzed a few endogenous microRNAs and observed that transfection with miR-328 did not affect expression of endogenous microRNAs (Figure S1B). Increased expression of *mir-328* was confirmed by real-time PCR (Figure 1C and Figure S2).

**Figure 2:**
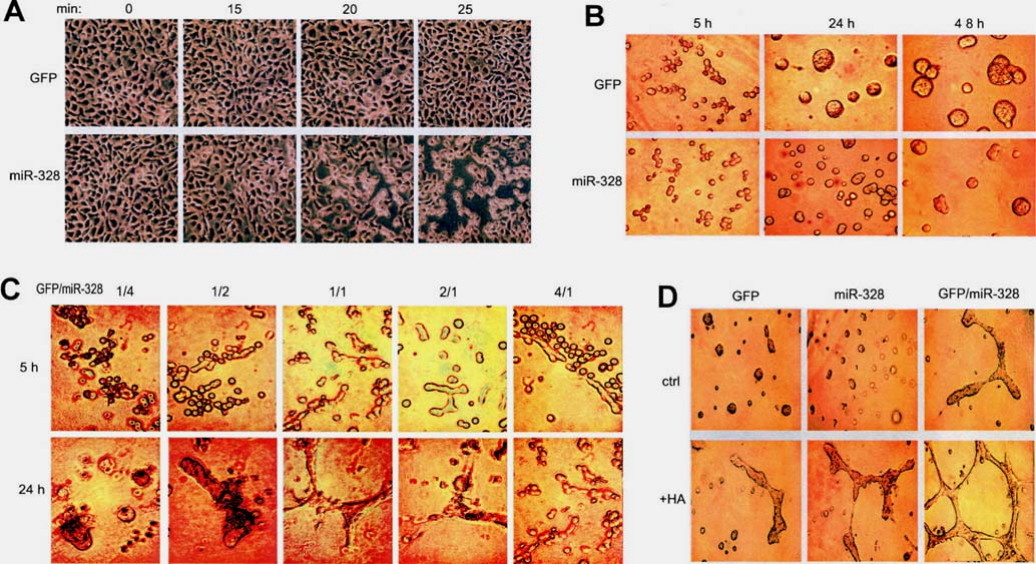
Expression of miR-328 reduces cell adhesion, aggregation, and capillary formation. (A) A431 cells transfected with miR-328 or a control vector were cultured in tissue culture plates to confluence. The cultures were treated with 10 mM EDTA for 30 min and recorded every 5 to 10 min. Cell detachment was examined under a light microscope and photographed. (B) GFP- and miR-328-transfected cells were incubated in Matrigel for 5, 24, and 48 hours. Reduction of cell aggregation was observed in the miR-328-transfected cells. (C) GFP- and miR-328-transfected cells were mixed in different ratios and cultured in Matrigel at 37°C for 5 or 24 hours followed by microscopic examination and photographed. Capillary formation reached the highest level when both groups of cells were mixed in a 1∶1 ratio. (D) GFP- and miR-328-transfected cells with or without mix (GFP/miR-328 in 1∶1 ratio) were cultured in Matrigel in the presence or absence of 150 µg/ml hyaluronan at 37°C for 24 hours. Addition of hyaluronan promoted the formation of capillary structure.

The effects of *miR-328* expression on cell activities were analyzed. A431 cells transfected with miR-328 (pooled cell line) or a control vector expressing GFP alone were cultured on tissue culture plates to confluence. The cultures were treated with 10 mM EDTA. Twenty min after the incubation, miR-328-transfected cells started to detach, while vector-transfected cells remained undetached after 25 min of incubation (Figure 2A). It appeared that *miR-328* expression might have suppressed some adhesion molecules on the cell surface. As a concordant activity to cell detachment is migration, wounding experiments were carried out to test migration activity of these cells. The experiments showed that the miR-328-transfected cells had a lower activity of migration (Figure S3A).

To examine how cell adhesion was affected, both GFP- and miR-328-transfected cells were incubated in the presence of manganese or calcium for 6 hours. Little difference was observed when manganese was present, suggesting that miR-328-promoted cell detachment was manganese-independent but calcium-involved (Figure S3B). Further experiments indicated that the effect of calcium was concentration dependent (Figure S3C-D). The cells were also incubated in tissue culture plates precoated without (ctrl) or with fibronectin (FN, 50 µg/ml), hyaluronan (HA, 5 mg/ml), and laminin (LN, 50 µg/ml) at 37°C for 2 hours followed by cell adhesion analysis. Reduction of cell adhesion was not observed in the plates precoated with laminin, suggesting that laminin was not involved in this process (Figure S4A).

To examine how these two groups of cells interacted with other extracellular matrix molecules, they were cultured in Matrigel (BD Matrigel basement membrane matrix, Cat No. 354234) for 5, 24, and 48 hours. Reduction of cell aggregation was observed in the miR-328-transfected cells (Figure 2B). This effect was growth factor-independent (Figure S4B, using BD growth factor reduced Matrigel basement membrane matrix, Cat No. 354263). Cell counting indicated that the formation of large complexes was not the results of proliferation (Figure S4C). Additional matrix molecules were also added to the Matrigel for aggregation assays. It showed that although addition of hyaluronan promoted aggregation of the GFP-transfected cells, expression of *miR-328* was able to reduce hyaluronan's effect on cell aggregation (Figure S5A). When hyaluronan reached a concentration of 100 µg/ml, cells expressing GFP alone formed branching-like structures and this was inhibited by *miR-328* expression (Figure S5B).

To test how these two groups of cells affected each other, they were mixed in different proportions and then cultured in Matrigel. When the cell mixture was at a ratio of 1∶1, formation of branching-like structures appeared (Figure 2C). Addition of hyaluronan promoted this process (Figure 2D), and growth factors were essential for the formation of branching-like structures (Figure S5C).

### Targeting of CD44 by *miR-328*

**Figure 3:**
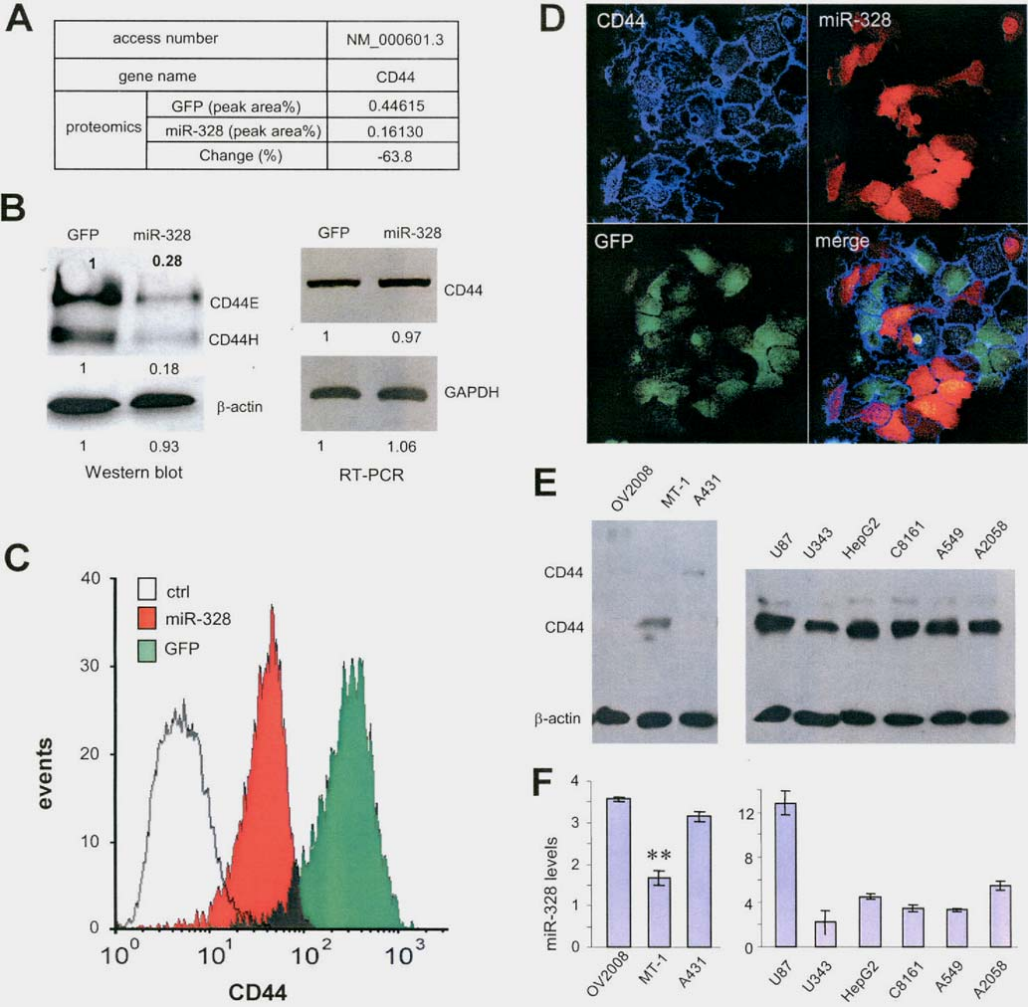
Targeting of CD44 by *miR-328*. (A) A431 cells transfected with miR-328 or a control vector were harvested and subjected to proteomic analysis. Repression of CD44 expression was observed. (B) Cell lysate from miR-328- or vector-transfected A431 cells was prepared and subjected to electrophoresis on a 10% SDS-polyacrylamide gel and analyzed by Western blotting for the expression of CD44. Repression of CD44 expression was observed in cells transfected with miR-328 (left). Staining for actin confirmed equal loading. RNA prepared from cells stably transfected with miR-328 and the control vector was subjected to RT-PCR (right) of CD44 (primers: huCD443*E*1917FSacI and huCD443*E*2680RMluI) and Gapdh (primers: huGapdh131F and huGapdh380R). (C) GFP- and miR-328-transfected cells were immunolabeled with anti-CD44 antibody followed by flow cytometry analysis. Transfection with miR-328 reduced CD44 expression. (D) Immunocytometry staining was performed in A431 cells stably transfected with miR-328/RFP or vector/GFP, followed by confocal microscopic examination of GFP (green), miR-328 (red), and CD44 expression (blue). Transfection with miR-328 repressed CD44 expression. (E) Protein samples were prepared from a number of human cell lines and subjected to Western blot probed with anti-CD44 antibody to analyze CD44 expression levels. Left, CD44 was expressed at relatively low levels. Right, CD44 was expressed at high levels. (F) RNA samples were prepared from the same cell lines and analyzed for *miR-328* expression by real-time PCR.

We then examined protein expression affected by *miR-328* transfection. A431 cells stably transfected with miR-328 or the vector were subjected to Western blot probed with a number of antibodies against different adhesion molecules including E-cadherin, P-cadherin, α-catenin, β-catenin, P-120, integrin α2, integrin α5, and integrin β1. No significant differences were detected between the cells transfected with miR-328 and cells expressing GFP alone (Figure S6A). The cells were also harvested and subjected to proteomic analysis performed by WEMB Biochem Inc (Richmond Hill, ON). A large number of proteins were down regulated by miR-328 transfection (Figure S7). One of them is CD44 (Figure 3A), the major receptor of hyaluronan [55], [56]. Western blot analysis confirmed that CD44 expression was repressed in the cells stably transfected with miR-328 (Fig 3B, left panel). RT-PCR assays showed little difference in the cells transfected with miR-328 as compared with the cells transfected with the control vector (Figure 3B, right panel). This suggests that *miR-328* represses CD44 expression at the translational level. To confirm the repression of CD44 by *miR-328* expression, both miR-328- and GFP-transfected cells were immunolabeled and analyzed by flow cytometry. A great reduction in CD44 expression was detected in the cells stably transfected with miR-328 as compared with the cells transfected with GFP (Figure 3C). Immunocytochemical staining further confirmed the repression of CD44 expression in cells transfected with miR-328 (Fig 3D). To confirm that the repression of CD44 expression was a consequence of *miR-328* expression, we generated a construct expressing an antisense RNA against *miR-328*. Transfection of the antisense construct enhanced CD44 expression (Fig S6B), which did not affect the level of mature *miR-328* (Fig S6C).

To test whether CD44 expression was correlated with *miR-328* levels, a number of human cell lines were analyzed for CD44 expression by Western blots (Fig 3E) and *miR-328* levels by real-time PCR (Fig 3F). We only detected an anti-correlation effect in three cell lines expressing low levels of CD44 (Fig 3E, left and Fig 3F, left). This anti-correlation effect was not observed in the cell lines expressing high levels of CD44 (Fig 3E, right and Fig 3F, right). In these cell lines, *miR-328* may not be the major regulator of CD44 since CD44 can also be regulated by many other miRNAs.

**Figure 4:**
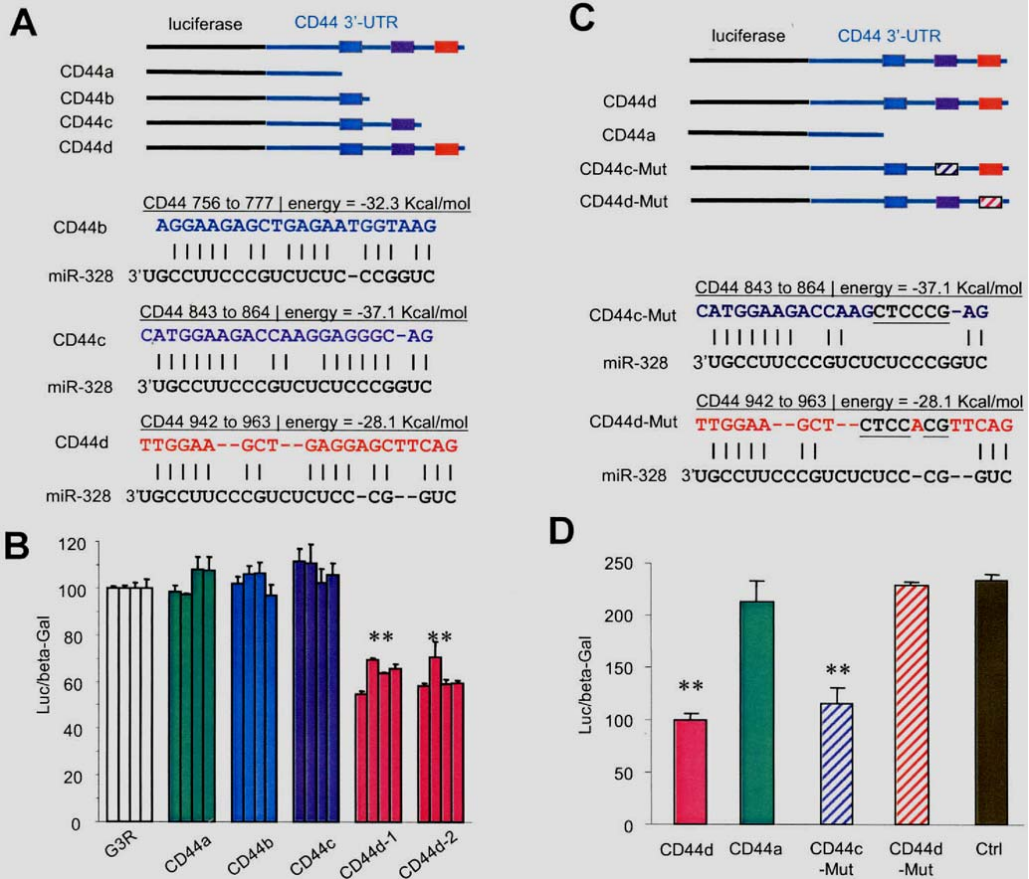
Confirmation of *miR-328* targeting CD44. (A) Computational algorithms were used to predict microRNAs that bind to a target sequence in the 3′-UTR of CD44. *miR-328* was found to have several potential target sites in CD44 3′-UTR. (B) COS-7 cells were co-transfected with the miR-328 and a luciferase reporter construct, which has been engineered with different fragments of the CD44 3′-UTR harbouring the target sequences of *miR-328* (Luc-CD44a, b, c, d). As a negative control, the luciferase reporter construct was engineered with a non-related fragment of cDNA (Ctrl). Significant differences are indicated by asterisks. **, p<0.01. Error bars, SD (n = 4). (C) Mutations were generated on the potential target sequences of CD44c and CD44d (underlined). (D) COS-7 cells were co-transfected with mature *miR-328* or RNA with random sequence (purchased from GenePharma, Shanghai) serving as a control (Ctrl) and a luciferase reporter construct, which has been engineered with a fragment of the CD44 3′-UTR harbouring the target sequences of *miR-328* (CD44d) or a mutant construct as indicated. Significant differences are indicated by asterisks. **, p<0.01. Error bars, SD (n = 4).

We then analyzed 3′-UTR sequence of CD44 and found three potential targets for *miR-328* (Figure 4A, GeneBank access number NM_001001390). To directly demonstrate repression of CD44 expression by *miR-328*, we integrated fragments of the CD44 3′-UTR containing the *miR-328* target sequences into a luciferase report vector (pMIR-Report, Ambion; for structures and sequence see Figure S8). Luciferase activity was significantly repressed in the construct harboring all the potential target sequences, as compared with the control vector harboring a non-related fragment (Ctrl), one potential target site, or two potential target sites (Fig 4B). This result suggests that the third sequence (CD44d, 5′ttggaagctgaggagcttcag) is essential for *miR-328* repression of CD44 expression. To confirm this, we generated mutations at this sequence (CD44d-Mut, 5′ttggaagctctccacgttcag) as well as in the potential sequence (CD44c) creating CD44c-Mut (5′catggaagaccaagctcccgag) in the luciferase reporter vector (Figure 4C). Luciferase activity assays indicated that mutation of the essential target site abolished the effect of *miR-328*, while mutation of the non-essential site produced little effect of *miR-328*'s repression activity (Fig 4D). These results further confirmed that the third sequence is essential for *miR-328* repression of CD44 expression. Although this sequence does not contain a “seed” sequence perfectly matched by *miR-328*, it receives a high score using the criteria we described recently [57].

**Figure 5:**
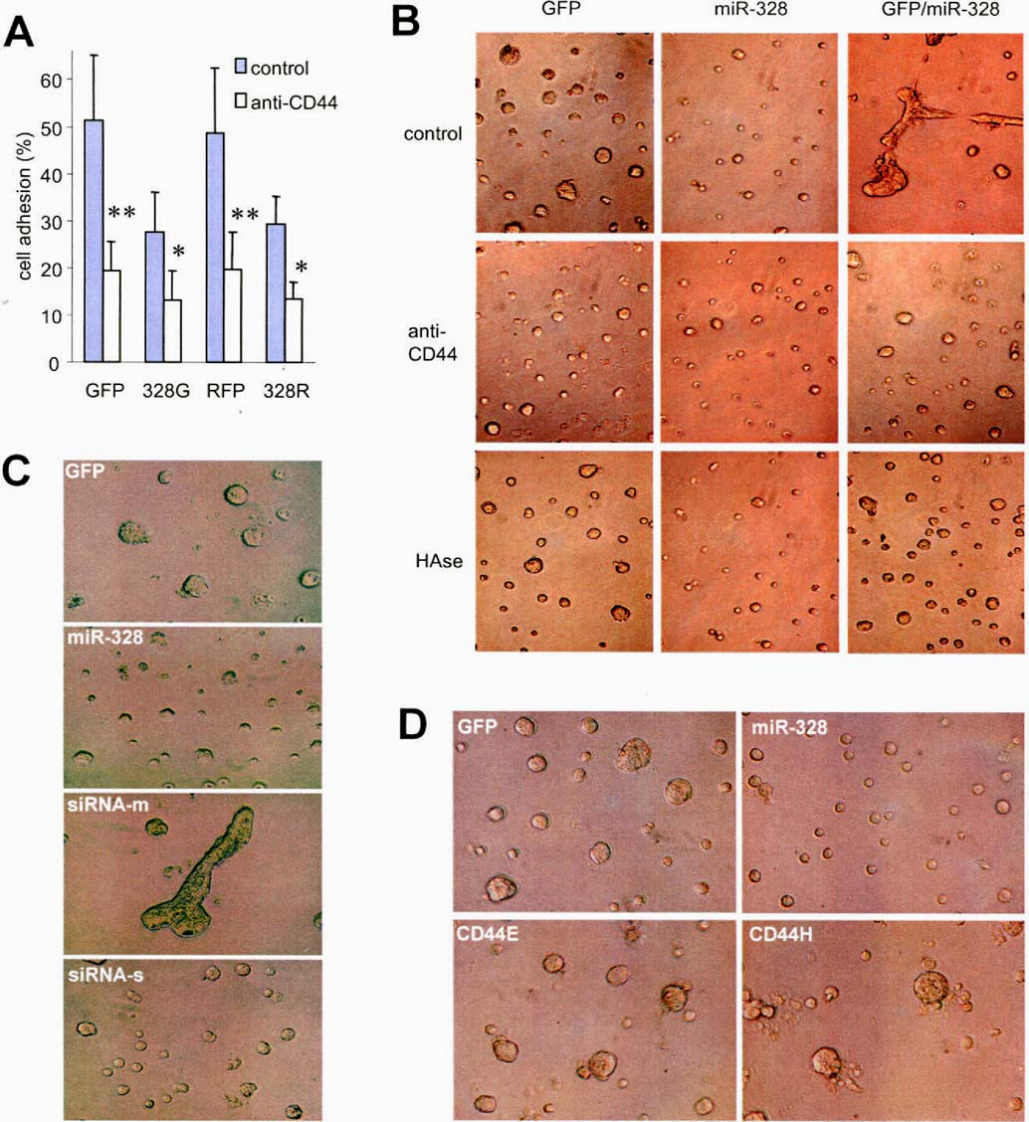
Rescue of *miR-328*-mediated cell activities. (A) GFP- and miR-328-transfected cells were incubated in tissue culture plates in the presence or absence of anti-CD44 antibody at 37°C for 2 hours followed by counting of the adherent cells. Addition of anti-CD44 antibody inhibited cell adhesion. Significant differences are indicated by asterisks. *, p<0.05. **, p<0.01. Error bars, SD (n = 5). (B) GFP- and miR-328-transfected cells without or with mix (GFP/miR-328 in 1∶1 ratio) were cultured in Matrigel in the presence or absence of hyaluronidase or anti-CD44 antibody at 37°C for 24 hours. Addition of hyaluronidase or anti-CD44 antibody inhibited cell aggregation and the formation of capillary structure. (C) A431 cells transiently transfected with an siRNA construct against CD44 were grown in Matrigel for 24 h. Partial reduction in CD44 expression, which contained a mix of transfected and non-transfected cells (siRNA-m) induced capillary formation compared to the GFP- or miR-328-transfected cells. The siRNA-transfected cells were then selected by cell sorting (siRNA-s) followed by culturing in Matrigel. Reduction in cell aggregation was observed. (D) A431 cells stably transfected with miR-328 or GFP vector were transiently transfected with CD44 or a control vector. Transfection of CD44 into the miR-328-expressing cells reversed the effect of *miR-328* in reducing cell adhesion.

To examine whether repression of CD44 levels was essential for *miR-328*-induced changes in cell activities, we used anti-CD44 antibody to block CD44's function in the miR-328-transfected cells. GFP- and miR-328-transfected cells were incubated in tissue culture plates in the presence or absence of anti-CD44 antibody at 37°C for 3 hours followed by counting of the adherent cells. Addition of anti-CD44 antibody inhibited cell adhesion (Figure 5A), but have little effect on cell survival, suggesting no cell toxicity of the reagents (Figure S9A). GFP- and miR-328-transfected cells with or without mix (in 1∶1 ratio) were also cultured in Matrigel in the presence or absence of hyaluronidase or anti-CD44 antibody at 37°C for 24 hours followed by microscopic examination and photographed. Addition of hyaluronidase or anti-CD44 antibody inhibited cell aggregation and formation of capillary structure (Figure 5B).

To corroborate this result, we generated a siRNA construct containing two hairpin structures complementary to CD44 sequences (Figure S9B, *Left*). Down regulation of CD44 was confirmed by Western blot (Fig S9B, *Right*). Cell aggregation was examined in A431 cells transfected with the siRNA construct or a control vector expressing GFP. The experiments indicated that transfection of CD44 siRNA promoted formation of the capillary-like structure (Fig 5C), since the transfection efficiency was approximately 20%, resulting in a mix of cells expressing high and low levels of CD44. When the transfected cells were enriched by sorting, inhibition of cell aggregation appeared in Matrigel culture, mimicking an effect of *miR-328*, suggesting that the CD44-pathway is essential for *miR-328*-mediated cell activities.

A431 cells stably transfected with miR-328 or GFP vector were transfected with CD44 or a control vector. Transfection was enriched by hygromycin selection. The cells were then cultured in Matrigel. Transfection of CD44 into the *miR-328*-expressing cells reversed the effect of *miR-328* in reducing cell aggregation (Figure 5D).

### Capillary formation affected by *miR-328* targeting CD44

**Figure 6:**
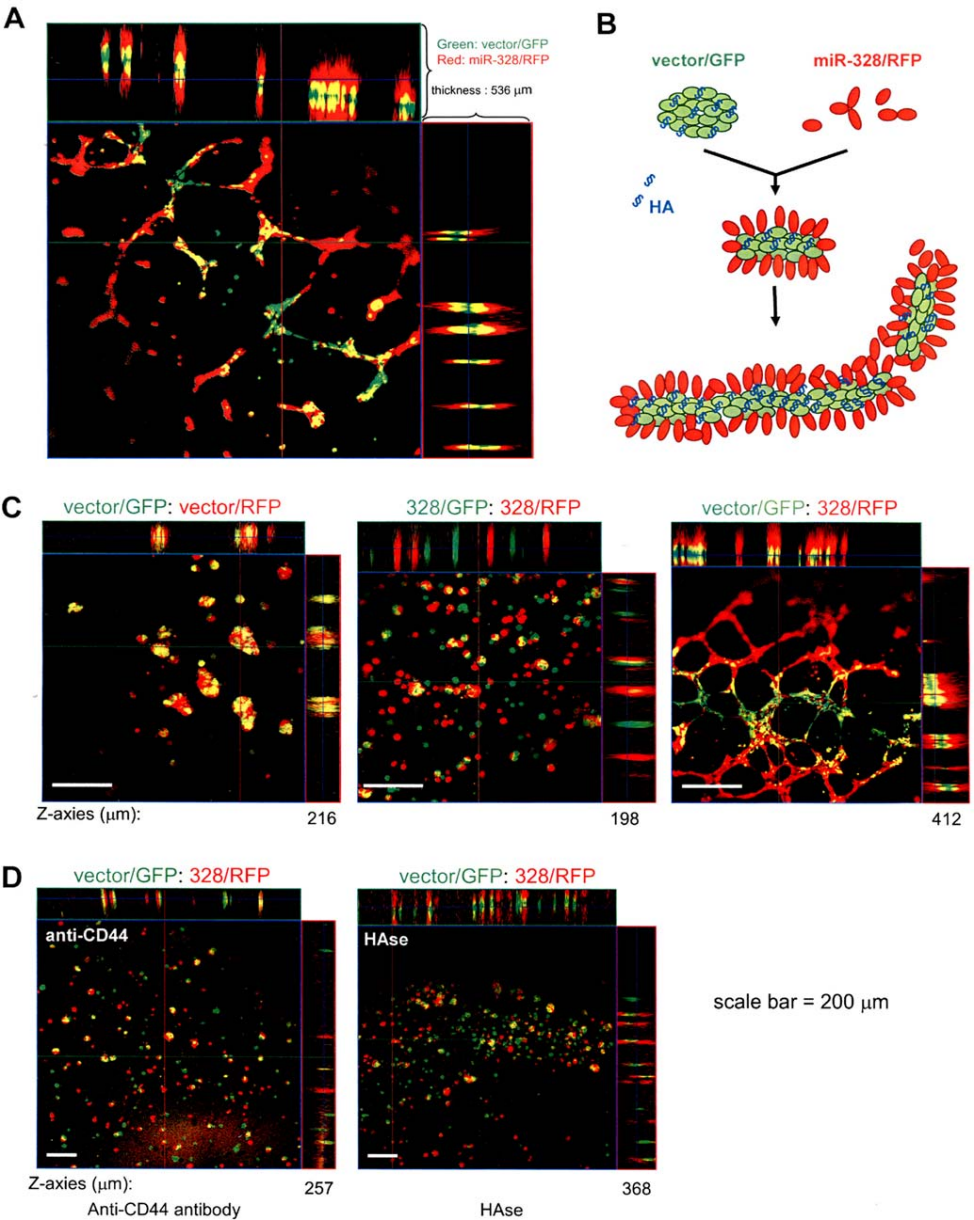
Capillary formation affected by *miR-328* targeting CD44. (A) GFP- and miR-328/RFP-transfected cells were mixed in a 1∶1 ratio and cultured in Matrigel at 37°C for 24 hours followed by confocal microscopic examination and photographed. Enlargement of a 536 µm-section exhibited the presence of green cells staying in the middle of the structures, while the red cells covering on the surface of the capillary structures. (B) A model of capillary formation affected by miR-328- and GFP-transfected cells. (C) Three types of cell mixture (1∶1) were obtained including: (i) cells transfected with vector/GFP mixed with cells transfected with vector/RFP, (ii) cells transfected with miR-328/GFP mixed with cells transfected with miR-328/RFP, and (iii) cells transfected with vector/GFP mixed with cells transfected with miR-328/RFP. The mixture was cultured in Matrigel for 24 hours. Random cell-cell interaction was seen in mix(i) and mix(ii). (D) Cells transfected with vector/GFP were mixed with cells transfected with miR-328/RFP with addition of anti-CD44 antibody or hyaluronidase (HAse). The mixture was cultured in Matrigel for 24 hours. Random cell-cell interaction was seen when the mixture was incubated with anti-CD44 antibody. Presence of red cells on the surface of green cells was seen when the mixture was treated with hyaluronidase.

To have a better insight of cell behaviour while CD44 expression was repressed by *miR-328*, Matrigel cultures of the mixture containing both the miR-328- and the GFP-transfected cells were subjected to confocal microscopic examination. The control cells, which expressed GFP alone (vector/GFP), always stayed in the middle of the tube-like structures, while the miR-328-transfected cells (328/RFP) always surrounded the tube-like structures (Figure 6A). Considering the presence of CD44 ligand hyaluronan, which enhanced cell aggregation, and the presence of E-cadherin, which mediated cell-cell interaction, we presented a model of cell-matrix-cell interaction based on the observation of the two groups of cell mixture (Figure 6B). Due to the repression of CD44, the miR-328-transfected cells could not form large complexes mediated by CD44-hyaluronan interaction. Nevertheless, the miR-328-transfected cells were able to interact with the vector/GFP cells because both groups of cells expressed equal levels of E-cadherin and, perhaps, some other adhesion molecules.

To confirm that the levels of CD44 expression affected cell localization, cells transfected with vector/GFP were mixed with cells transfected with vector/RFP, both of which expressed equally high levels of CD44 (data not shown). The mixture was cultured in Matrigel. Confocal microscopic examination indicated that both groups of cells randomly mixed with each other and formed large complexes (Figure 6C, vector/GFP:vector/RFP). Cells transfected with miR-328/GFP were mixed with cells transfected with miR-328/RFP, both of which expressed equally low levels of CD44 (data not shown), followed by Matrigel culturing. Confocal analysis indicated that the cells mixed with each other randomly and formed small complexes (Figure 6C, miR-328/GFP:miR-328/RFP). Formation of tube-like structures could only be seen when vector/GFP-transfected cells were mixed with miR-328/RFP-transfected cells, in which the green cells stayed in the middle while the red cells covered the surface of the tube-like structures.

To confirm the function of CD44 in the formation of tube-like structures, the mix containing cells transfected with vector/GFP and cells transfected with miR-328/RFP was incubated with anti-CD44 antibody. The tube-like structures were no longer formed (Fig 6D). Instead, both groups of cells randomly interacted with each other, and small complexes were obtained.

To test how hyaluronan affected the formation of the tube-like structures, the mix containing cells transfected with vector/GFP and cells transfected with miR-328/RFP was incubated with hyaluronidase. The tube-like structures were not formed, and only small complexes were obtained (Figure 6D). However, the vector/GFP-transfected cells still stayed in the middle while the red cells covered on the surface of the small complexes, indicating that hyaluronidase could only disrupt the formation of the tube-like structures but could not change the location of the cells. It also suggested that high-and-low levels of CD44 are essential for the formation of zonating structures, implying a role of CD44 in tissue differentiation. This is in agreement with a previous report that CD44 mediates cell differentiation [58].

### Zonating morphogenesis affected by *miR-328* targeting CD44 *in vivo*

**Figure 7:**
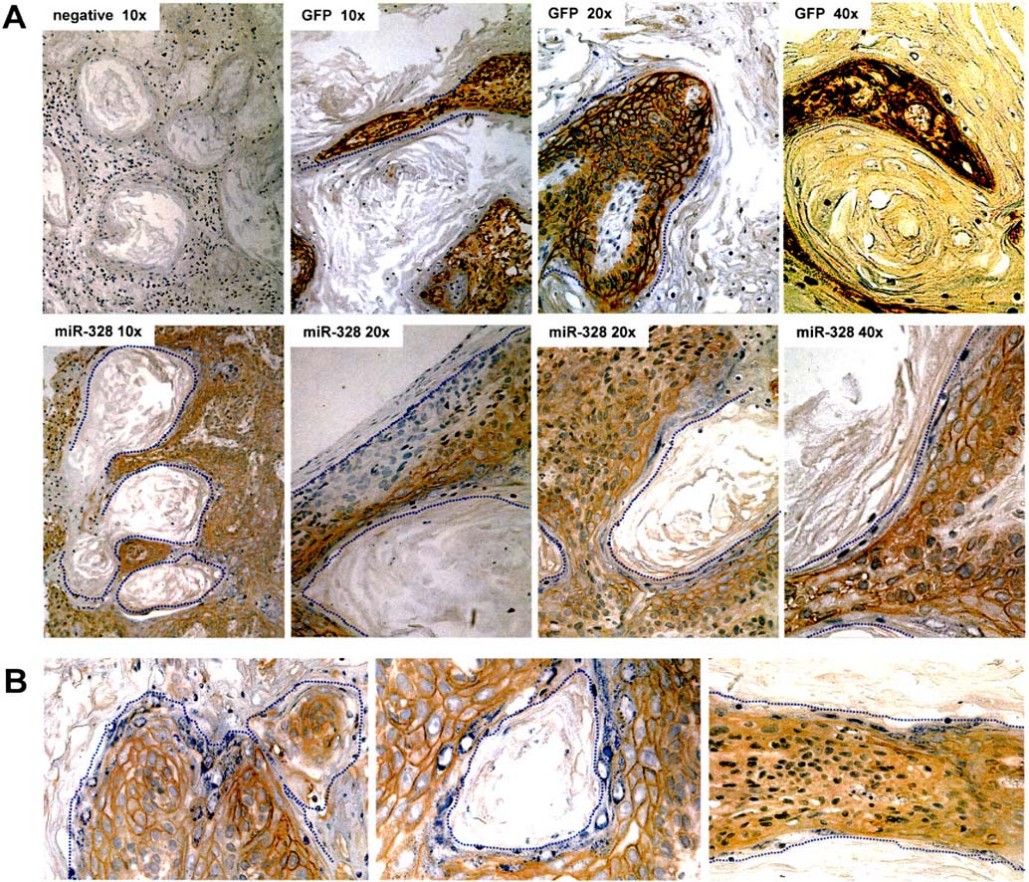
Zonating morphogenesis affected by *miR-328* targeting CD44 in nude mice. (A) Tumors formed by injection of cells transfected with GFP or miR-328 were sectioned, immunostained for CD44, and photographed at 10x, 20x, and 40x magnifications. As a negative control, section was stained with secondary antibody alone. In the miR-328 group, layers of CD44-negative cells (blue nuclei) were detected between tumor tissues and stroma tissues. This was not evident in the GFP group, which expressed high levels of CD44. The dotted lines mark the boundary between the tumors and stroma tissues. For those areas in which the boundary is clear, no dotted line was provided. (B) Magnified photos showing the boundary between tumor tissues and stroma tissues. The CD44-negative cells were able to cover the surface of nodules (left), to form a zonating layers inside the tumor tissue (center), or to cover the surface of column-like structures (right).

We then used an *in vivo* model to test the effects of CD44 on cell-cell interaction. The miR-328- and GFP-transfected A431cells were injected subcutaneously into nude mice. Four weeks after the injection, the mice were sacrificed and tumors were removed. The tumors were sectioned, immunostained for CD44. Given their epithelial origin, the melanoma A431 cells mixed well with the stroma tissues and formed multiple small nodules. In the group injected with miR-328-transfected cells, layers of CD44-negative cells (blue nuclei) were detected between tumor tissues and stroma tissues, while the CD44-positive cells were largely detected in the internal locations of the tumors (Fig 7A). On the other hand, in the GFP-transfected cells there were little or no CD44-negative cells in the boundary between the tumors and the stroma tissues, since these cells expressed high levels of CD44. It was observed that some cells with blue nuclei were CD44-negative, which were either mixed with stroma cells or surrounding the tumor tissues. Some photos with large magnifications are provided to show that the CD44-negative cells always like to push the CD44-positive cells together, forming column-like or zonating structures (Fig 7B).

It was noted that the mouse stroma cells were mainly CD44-negative, since the antibody (sc-9960 from Santa Cruz Biotechnology), which recognizes both human and mouse CD44, did not exhibit clear staining of CD44 in the stroma cells (data not shown). This may be the reason why A431 cells cannot form large tumors in the skin location, as the stroma cells may be able to guild the tumor cells to form certain smaller structures. Although the CD44-positive cells can potentially form large tumors, as there is an advantage for the same type of cells to assemble together for survival, the CD44-negative stroma cells seemed not to allow this to occur and were able to facilitate the formation of certain shape of structures guided by the stroma cells. In the case of *miR-328*-expressing cells, the levels of CD44 were low and some were negative. Although these CD44-negative cells now served as the guide cells for CD44-positve cells (low levels of CD44) to form some tumor structures, they would not push the CD44-positive as hard as the stroma cells did. As a result, the miR-328-transfected cells were able to form relatively larger tumor nodules than the GFP-transfected cells did. The fact that cells expressing high levels of CD44 formed more but smaller nodules suggests that high levels of CD44 would promote tumor metastasis. This is in agreement with previous reports that CD44 plays a role in cancer metastasis [59]. We also examined CD44 expression on sections of tumors formed by astrocytoma cell line U87 and found that the surface layers of the tumors were CD44-negative while the internal tumor sections expressed very high levels of CD44 (Fig S9C).

**Figure 8:**
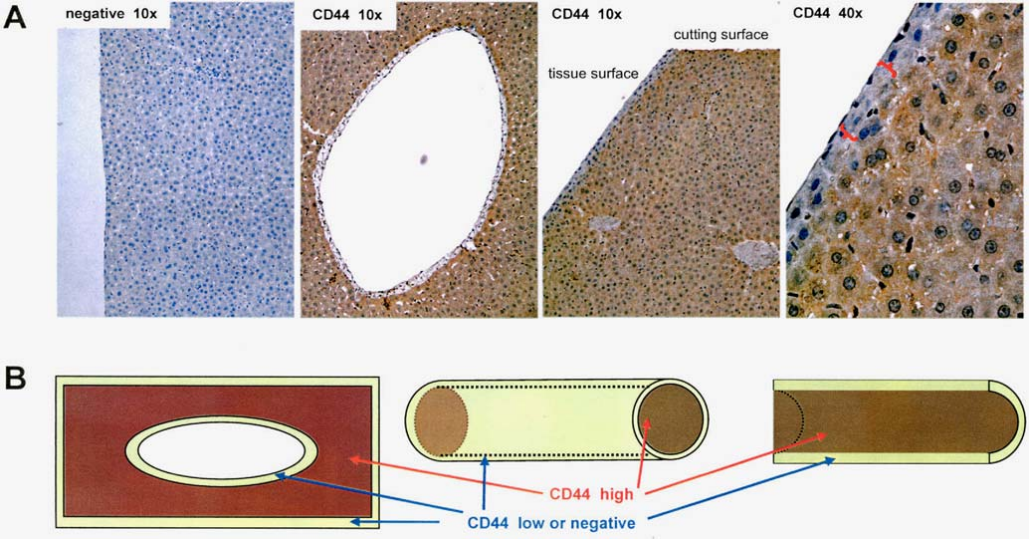
Zonating morphogenesis associated with CD44 expression in rat liver. (A) Rat livers were sectioned and immunostained with anti-CD44 antibody or the secondary antibody alone. CD44 expression was detected throughout the liver tissues. Note that the surface layers of cells as indicated by the brackets and the internal zones of the Portal veins were CD44 negative. (B) A model showing CD44 expression patterns.

We then examined whether CD44 expression is low or negative on the surface of a naturally existing organ or tissue structure. It was noted that the cell types on the surface and the internal region of an organ should be the same to allow comparison. Liver is such an ideal organ in that the cells on the surface and the internal of a functional unit are the same, namely the hepatocytes. Rat livers were sectioned and immunostained with anti-CD44 antibody. CD44 expression was detected throughout the liver tissues. However, the surface layers of cells of the tissue and the internal zones of the Portal veins were CD44 negative (Fig 8A). It seems that covering of CD44-positive cells with CD44-negative cells is a requirement for tissue formation.

Our *in vitro* and *in vivo* results show that CD44-negative cells function as a barrier for CD44-positive cells to expand: these CD44-negative cells sealed the surface of the tissues. Only when there were no CD44-negative cells, could the CD44-positive cells extend. Our results explain why CD44 is always highly expressed in the developing distal tips of epithelial ducts where it binds to hyaluronan in the growing buds [60]. As tissues become mature, CD44 expression decreases [61]. Each organ seems to have particular mechanisms to form the shape of its preference, and regulation of CD44 expression appears to be one of them. We have thus proposed a CD44 expression model for zonating morphogenesis (Fig 8B).

In this study, we have demonstrated that a construct harbouring a duplicate of pre-microRNA miR-328 can be successfully expressed and processed to mature microRNA. With this approach, we have demonstrated that cells expressing *miR-328* exhibited reduced adhesion and formed small complexes in Matrigel. We also found that CD44 is the target of *miR-328* in mediating a variety of cell activities. Our research now paves the way to understand the roles of microRNAs in an unexplored area of development.

## Materials and Methods

### Materials and Cell Culture

Anti-CD44 (H3) antibody was a gift from Dr. Liu Cao. Anti-actin and HRP-conjugated secondary antibodies were purchased from Sigma. Cy5-conjugated secondary antibody was purchased from Abcam. Other antibodies were purchased from Chemicon (integrin α5), Santa Cruz Biotechology (integrin β1), BD Transduction Laboratories (E-cadherin and P-cadherin) and Zymed Laboratories (α-catenin and β-catenin). Matrigel (basement membrane matrix and growth factor reduced) were purchased from BD Transduction Laboratories. Luciferase assay kits were purchased from Promega. *o*-nitrophenyl-β-D-galactopyranoside (ONPG) was purchased from Calbiochem. Dulbecco's modified Eagle's medium (DMEM), fetal bovine serum (FBS), Hank's balance salt solution (HBSS), trypsin/EDTA, Lipofectamine, Geneticin (G418), hygromycin, oligonucleotides, T4 DNA ligase, platinum *Pfx* DNA polymerase, SuperScript II reverse transcriptase, random primer and DH5α were purchased from Invitrogen. Restriction enzymes were purchased from New England Biolabs. REDTaq DNA polymerase and other materials were purchased from Sigma.

### Construct Generation

A microRNA construct expressing *miR-328* was designed by our lab and the DNA synthesized by a biotech company (Top Gene Technologies, Montreal). In brief, the pre-microRNA of *miR-328* was ligated into a mammalian expression vector BluGFP that contains a Bluescript backbone, a CMV promoter driving expression of either green fluorescent protein (GFP) or the red fluorescence protein (RFP), and a H1 promoter driving *miR-328*. This plasmid was developed in our lab and is expected to simultaneously express a small fragment of RNA and produce GFP (Fig 1A). This means that every fluorescent cell expresses mature *miR-328*. Using similar approach, an antisense sequence to *miR-328* was inserted in the expression vector producing an anti-*miR-328* construct. We have used this method to produce pre-miRNAs and mature miRNAs [40].

The siRNA construct against CD44 was made by primers of huCD44-si868p1 and huCD44-si1115p2 and was ligated with a CMV promoter driving the green fluorescence protein (GFP) into the mammalian expression vector (Shown as Supplementary Figure S9B).

A luciferase reporter vector (pMir-Report; Ambion, Austin, TX) was used to generate luciferase reporter constructs. A fragment of the 3′-untranslated region (3′-UTR) of human CD44 was cloned with two primers, huCD443*E*1917FSacI and huCD443*E*2884RMluI, by RT-PCR. The PCR product was digested with SacI and MluI and inserted into a SacI- and MluI-opened pMIR-REPORT Luciferse plasmid (Ambion), obtained a construct named Luc-CD44-d (Fig S8). Three more fragments containing continuing deletion were generated by using huCD443*E*1917FSacI combined with huCD443*E*2680RMluI, huCD443*E*2697RMluI, or huCD443*E*27884RMluI in PCR. Digestion and ligation of these products led to the production of three constructs named Luc-CD44-a, Luc-CD44-b, and Luc-CD44-c (Fig S8). To serve as a negative control, a non-related sequence was amplified from the coding sequence of the chicken versican G3 domain using two primers chver10051-SpeI and chver10350-SacI. It is expected that no endogenous microRNA would target this fragment as it is located in the coding sequence. The amplified PCR product was digested with SpeI and SacI, followed by insertion into a SpeI- and SacI-open pMir-Report vector. Primers huCD443*E*1917FSacI and huCD44-2884R-mu were used to generate mutation construct CD44d-Mut. To generate mutation construct CD44c-Mut, a two-step PCR was performed. The first PCR used primers huCD443*E*1917FSacI and huCD44-2833Rmu followed by a second PCR using primers huCD443*E*1917FSacI and huCD44-2884R. The PCR product was digested with SacI and MluI, followed by insertion into a SacI- and MluI-open pMir-Report vector. The protein expression constructs, CD44E and CD44H, were kind gifts from Dr. Warren Knudson [62].

The identities of all constructs were confirmed by restriction digestion and sequencing.

To confirm expression of different constructs, control vector or miR-328 with GFP or RFP was transfected into A431 cells by lipofatamine 2000, and selected by G418 or hygromycin for getting stable cell lines. Then, cells were further sorted by GFP or RFP. Cells were maintained in DMEM with 10%FBS.

### RT-PCR and RNA Analysis

For pre- and mature microRNA concentration assays, 10^7^ cells were harvested for RNA isolation. Small RNA (<200 nt) enriched total RNA was isolated by mirVana miRNA isolation kit (Ambion). After the adjustment of RNA concentration, 3% agarose gel was used to check the RNA quality and amount. 1 µg RNA was used for reverse transcription with Superscript II reverse transcriptase. The three primers, RT-328-2, PCR-328-2, PCR-miRNA-3, were designed for the reverse transcriptase-PCR (RT-PCR) of mature *miR-328*. For the pre-miR-328 assay, random primer was used for reverse transcription, than primer miR-328N and miR-328C were used for PCR. Gapdh (primers: huGapdh131F and huGapdh380R) was used as a control. PCR was carried out at the temperature of 94°C, 56°C, and 72°C of 25 cycles.

For the mRNA level of CD44, total RNA from 3×10^6^ cells was isolated by RNeasy mini kit (Qiagen). After the adjustment of RNA concentration, 1% agarose gel was used to check the RNA quality and amount. 1 µg RNA was used for reverse transcription with Superscript II reverse transcriptase. Random primer (Invitrogen), primer huCD443*E*1917FSacI, and huCD443*E*2680RMluI were used for RT-PCR. Gapdh (primers: huGapdh131F and huGapdh380R) was used as a control. PCR was carried out at the temperature of 94°C, 56°C, and 72°C of 25 cycles as described previously [63].

### Western Blotting

Cells were seeded onto a 6-well plate at 10^6^ cells/well. Proteins were extracted 24 hours after plating by lysing the cells in each well in 100 µl of lysis buffer containing protease inhibitors (150 mM NaCl, 25 mM Tris-HCl, pH 8.0, 0.5 M EDTA, 20% Triton X-100, 8 M Urea, and 1x protease inhibitor cocktail). After the addition of lysis buffer, the cells were scraped off and keep on ice for 30 min. Protein concentration of lysates were measured by Bio-Rad protein assay kit after centrifugation at 14000 rpm for 10 min. Lysates with 30 µg of protein were subjected to SDS-PAGE for CD44 detection. Lysates with 40∼80 µg of protein were subjected to SDS-PAGE for the detection of other adhesion molecules. The separated proteins were transferred to nitrocellulose membranes followed by immunostaining with a primary monoclonal antibody overnight at 4°C. After washing, the membrane was incubated with appropriate HRP-conjugated secondary antibody for 1 hour at room temperature followed by ECL detection. After detection of protein bands, the blot was re-probed with the primary antibody against β-actin to confirm equal loading of samples. The monoclonal β-actin antibody (Sigma-Aldrich) was incubated with the blot for 2 hours at room temperature at a dilution of 1∶5000. The secondary antibody used was once again the goat anti-mouse IgG (1∶2000) for 1 hour at room temperature, followed by washes, detection with the ECL kit, and autoradiography.

### Cell Adhesion Assay

Petri-dishes were coated with 50 µg/ml laminin, fibronectin or 5 mg/ml hyaluronic acid for one hour and washed by PBS. For ECM protein binding assay, 10^6^ cells/ml in DMEM+10%FBS were seeded at 3.5 cm coated or uncoated petri-dishes for 3 hours. For ion adhesion assay, 10^6^ cells/ml in HBSS with calcium or manganese in different concentration were seeded at 3.5 cm uncoated Petri dishes for 6 hours. Adhering cells were counted. Eight different fields (100x) in three dishes were used for calculation (n = 8±SD).

### Cell Aggregation Assay

4×10^4^/cm^2^ cells were seeded in BD Basic Matrixgel (basement membrane matrix or growth factor reduced) with DMEM+10%FBS for 24 or 48 hours. Exogenous anti-CD44, hyaluronan or hyaluronidase were added in different experiments. The images were captured in bright field using a light microscope or a confocal microscope in a z-section program.

### Assays of Luciferase Activity

Luciferase activity assays were performed using the Promega Luciferase Assay System (Madison, WI) using the methods described [54]. In brief, COS-7 cells (10^5^ cells/well) were seeded onto 12-well tissue culture plates overnight. The cultures were changed for serum-free medium prior to transfection with different combinations of DNA (e.g. 10 ng luciferase reporter construct mixed with 10 ng β-Gal plasmid and 300 ng miR-328 construct). Twenty hours after transfection, the transfection reagent was replaced with DMEM+10%FBS culture medium, and the cultures were maintained at 37°C for 24 hours. Cells were then harvested using trypsin/EDTA and lysed with 150 ml 1x lysis buffer diluted from luciferase 5x lysis buffer on ice for 30 min. After centrifugation at 14000 rpm for 15 seconds, the supernatant was used to measure β-Gal activity and luciferase activity in 96-well plates in triplicate using the Promega Luciferase Assay System in TopCount NXT (a microplate scintillation and luminescence counter produced by Packard Instrument Company, Meriden, CT). For the luciferase activity assay, 10 µl/well of the supernatant was mixed with 70 µl/well of Luciferase Assay Substrate A in black plates in a dark room followed immediately by reading of the luciferase activity. For the β-Gal activity assay, 50 µl/well of the supernatant was mixed with 90 µl/well of Substrate B (*o*-nitrophenyl-β-D-galactopyranoside or OPNG) in transparent plates followed by incubation at 37°C for 1 hour before reading. The experiments were repeated four times. Luciferase activities were normalized against β-Gal activity.

### Flow Cytometry Analysis and Immunocytochemistry

For flow cytometry detection, 10^5^ cells/well were seeded into 12-well plates for overnight culture then harvested by 10 mM EDTA in PBS. For immunocytochemistry, control cells with GFP and miR-328 transfected cells with RFP were mixed together in a ratio of 1∶1 and seeded into 12-well plates with cover slices in a concentration of 5×10^4^ cells/well. The cells were fixed in 3.7% paraformaldehyde at room temperature for 30 min blocked with 1% BSA in PBS. They were then immunostained with primary antibody diluted in blocking solution at room temperature for 1 hour. 2 µg/ml anti-CD44 and 1 µg/ml cy5-conjugated secondary antibody in PBS with 1% BSA were used for staining. The cells were rinsed three times with PBS and incubated with secondary antibody coupled to cy5 for 45 min. Slides were then rinsed three times with PBS before mounting, and visualized using a Zeiss confocal microscope using methods described [64].

### Tumorigenicity Assays in Nude Mice and Immunohistochemistry

The methods have been described by us previously [65], [66]. In brief, six-week-old nude mice strain Balb/c were injected with miR-328- and GFP-transfected U87 cell lines (5×10^6^ cells in 100 PBS). Tumors were measured weekly thereafter. Tumor volume (V) was measured using a caliper by determining the length (L) and width (W), where V = (L×W^2^)/2. Four weeks after injection, tumors were removed for further analysis. To confirm the effect of *miR-328*, we used a different cell line for tumorigenesis assay each time. Tumors were fixed in 10% buffered formalin, processed, and embedded in paraffin. Immunohistochemistry was performed on 5 µm paraffin sections mounted on charged slides. The sections were stained with hematoxylin and eosin (H&E) and immunostained with either anti-CD44 antibody or secondary antibody as a control using the ABC and DAB Kits from Vector Laboratories Inc (Burlingame, CA).

### Statistical Analysis

The results (mean values±SE) of all the experiments were subjected to statistical analysis by *t*-test. The level of significance was set at p<0.05.

## Supporting Information

Table S1 (0.04 MB DOC)

Figure S1 A, Diagram of the procedure of RT-PCR of mature microRNA miR-328. The mRNA was isolated with mirVana miRNA Isolation Kit (Ambion, Austin, TX). RT-PCR was performed using Superscript II Reverse Transcriptase (Invitrogen). PCR was carried out at the temperature of 94°C, 56°C, and 72°C for 25 cycles. B, RT-PCR of mature miR-328, miR-378, miR-17-3p, and miR-17-5p using RNA prepared from A431 cells stably transfected with miR-328 and a control vector, confirming that processing of other microRNAs was not affected by miR-328 transfection.
(0.12 MB PPT)

Figure S2 A, The melting curves in real-time PCR experiments reveal undetectable contamination, mispriming, and primer-dimer artifacts of all samples indicated on the left of the curves. B, Real-time PCR curves of mature miR-328 from RNAs isolated from miR-328- and GFP vector-transfected cells. Real-time PCR was carried out according to the manufacturer's instructions (Qiagen, miScript Reverse Transcription Kit, cat #218060; miScript Primer Assay, cat #218411; miScript SYBR Green PCR Kit, cat #218073).
(0.23 MB PPT)

Figure S3 A, GFP- and miR-328-transfected cells were cultured on tissue culture plates to confluence. The cultures were wounded by a micro-tip. Migration of cells to the wounding areas was examined under a light microscope and photographed. [Cell migration assay: Wound healing experiments were used to examine cell migration. Cells (3×106) were seeded on 3.5 cm culture dishes in DMEM supplemented with 10% FBS. Twenty-four hours after cell inoculation, sub-confluent monolayers were wounded linearly by scraping with p1000 pipette tips, washed to remove cell debris and refilled with fresh media. The images were captured at the beginning and 15 hours later with a phase-contrast microscope.] B, GFP- and miR-328-transfected cells were incubated in Petri dishes in the presence of HBSS with manganese (Mn) or calcium (Ca) for 6 hours. C, GFP- and miR-328-transfected cells were incubated on Petri dishes in the presence of 0.2, 0.5, and 1.0 mM CaCl2 for 6 hours. Cell adhesion was analyzed by counting the cells that adhered to plates. miR-328-transfected cells exhibited lower rates of adhesion than the GFP-transfected cells. Error bars, SD (n = 8). D, typical micrographs of cell attachment are shown.
(2.64 MB PPT)

Figure S4 A, Expression of miR-328 reduces cell adhesion. GFP- and miR-328-transfected cells were incubated in Petri dishes precoated without (Ctrl) or with fibronectin (FN, 50 mg/ml), hyaluronan (HA, 5 mg/ml), and laminin (LN, 50 mg/ml). The cultures were maintained at 37°C for 3 hours in culture medium followed by microscopic examination. Adherent cells were counted. Reduction of cell adhesion was observed in the miR-328-transfected cells. B, FP- and miR-328-transfected cells were incubated in Matrigel with or without growth factors. C, cell number was counted to determine rates of proliferation. [Proliferation assay: 2×105 /well of vector- and miR-328-transfected A431 cells were seeded to 6-well plastic tissue culture plates in DMEM containing 10% FBS, and incubated for different time periods. The cultures were trypsinised and concentrated to a volume of 100 nl for counting by hemocytometer (n = 3±S.D.).] μ
(1.12 MB PPT)

Figure S5 A, GFP- and miR-328-transfected cells were incubated in Matrigel without (Ctrl) or with hyaluronan (HA, 50 ng/ml), fibronectin (FN, 50 ng/ml), and laminin (LN, 50 ng/ml). The cultures were maintained at 37°C for 24 hours followed by microscopic examination and photographed. Reduction of cell aggregation was observed in the miR-328-transfected cells. B, The cells were also incubated in Matrigel with the addition of 0, 25, 50, and 100 mg/ml hyaluronan for 24 hours. Addition of hyaluronan promoted tube-like structure formation. C, GFP- and miR-328-transfected cells were mixed in a 1:1 ratio. The mixed cells were incubated in Matrigel with or without growth factors (GF) at 37°C for 24 hours followed by microscopic examination and photographed. Presence of growth factors promoted the formation of tube-like structures. μ
(7.07 MB PPT)

Figure S6 A, GFP- and miR-328-transfected cells were cultured on tissue culture plates to confluence. Cell lysate was prepared and subjected to Western blotting probing with antibodies against different adhesion molecules as indicated. Little difference was detected. B, GFP-, miR-328-, and anti-miR-328 (antisense against miR-328)-transfected cells were cultured on tissue culture plates to confluence. Cell lysate was prepared and subjected to Western blotting probing with antibodies against CD44 or actin. C, RNA samples were also prepared for RT-PCR amplifying pre-miR-328 and mature miR-328.
(0.99 MB PPT)

Figure S7 Proteomic results. GFP- and miR-328-transfected cells were cultured on tissue culture plates to confluence. Cell lysate was prepared and subjected to proteomic analysis. A number of proteins were detected to be down-regulated (A) and up-regulated (B) by miR-328-transfection.
(2.30 MB PPT)

Figure S8 Luciferase report constructs for CD44. Fragments of CD44 3'UTR were inserted into the luciferase report vector pMiR-Report producing a constructs named Luc-CD44a, Luc-CD44b, Luc-CD44c, and Luc-CD44d . The potential mir-328 target sequences were labelled in blue. A control sequence (Ctrl) was obtained from the G3 domain of chicken versican.
(0.04 MB PPT)

Figure S9 A, GFP- and miR-328-transfected cells were cultured in the presence or absence of anti-CD44 antibody or hyaluronidase at 37°C for 48 hours followed by viability assays using an MTT assay kit. (Toxicity assay: Cells were seeded at a concentration of 2×103 cells/well in 96-well plate without or with anti-CD44 antibody or hyaluronidase for 48 hours. MTT assay was used for detecting cell viability.) B, siRNA construct for CD44 (left). siRNA-mediated silencing of CD44 was examined by Western blot probed with anti-CD44 antibody (right). C, Tumor tissue stained with CD44. Tumors formed by astrocytoma cells U87 were subjected to immunohistochemistry with anti-CD44 antibody. CD44-negative layers as indicated by the brackets were detected between the tumor tissue and the stroma tissue.
(1.13 MB PPT)
