# Correction: MicroRNA miR-328 Regulates Zonation Morphogenesis by Targeting CD44 Expression

**DOI:** 10.1371/journal.pone.0362015

**Published:** 2026-11-04

**Authors:** Chia-Hui Wang, Daniel Y. Lee, Zhaoqun Deng, Zina Jeyapalan, Shao-Chen Lee, Shireen Kahai, Wei-Yang Lu, Yaou Zhang, Burton B. Yang

There is an error in Fig 2C in this article [1]: the 5 h 1/1 panel in Fig 2C was duplicated in the 24 h 4/1 panel in Fig 2C.

The corrected Fig 2 is provided with this notice, and the image data provided in support of this figure are in S1-S10 Files.

**Fig 2:**
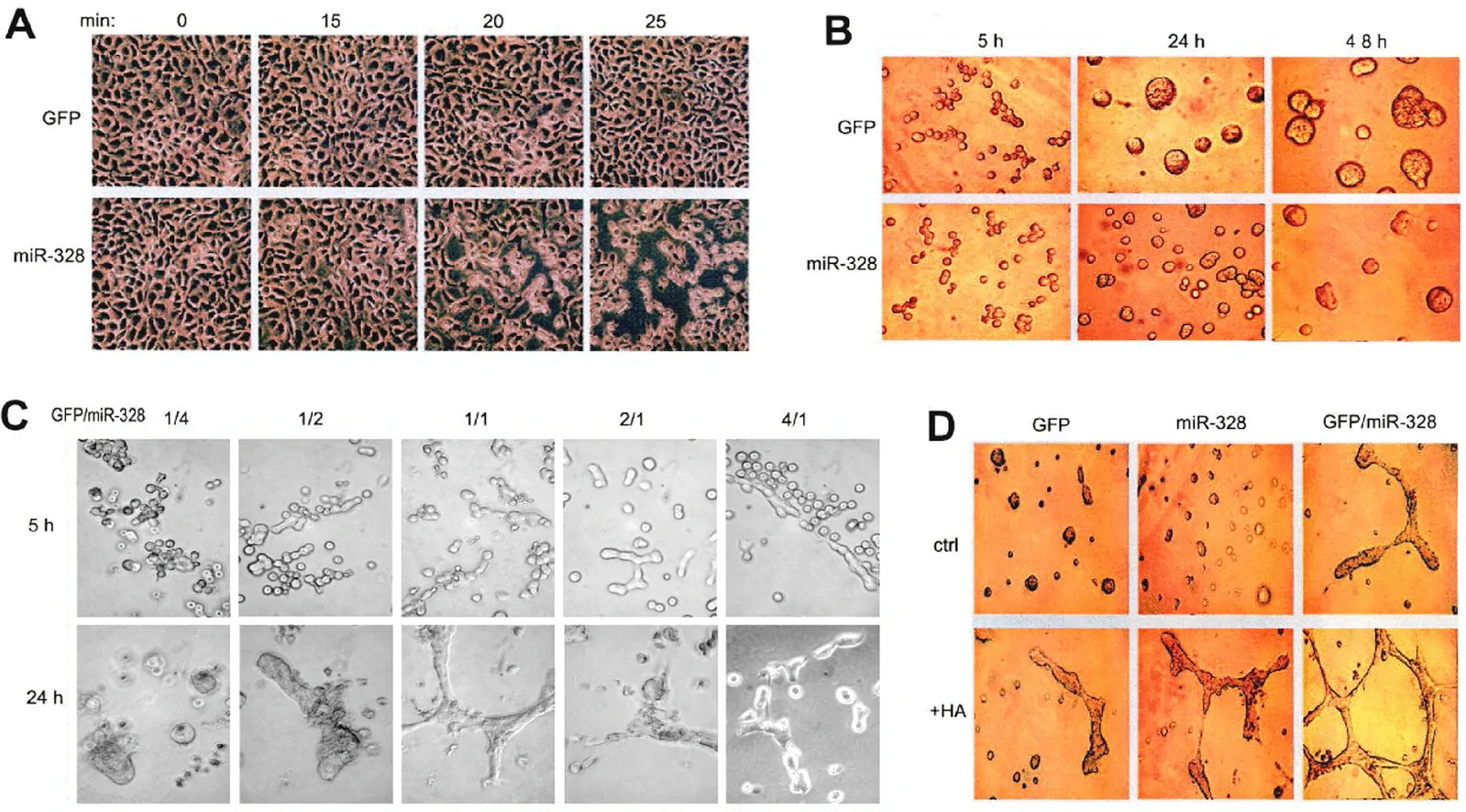
Expression of miR-328 reduces cell adhesion, aggregation, and capillary formation. (A) A431 cells transfected with miR-328 or a control vector were cultured in tissue culture plates to confluence. The cultures were treated with 10 mM EDTA for 30 min and recorded every 5 to 10 min. Cell detachment was examined under a light microscope and photographed. (B) GFP- and miR-328-transfected cells were incubated in Matrigel for 5, 24, and 48 hours. Reduction of cell aggregation was observed in the miR-328-transfected cells. (C) GFP- and miR-328-transfected cells were mixed in different ratios and cultured in Matrigel at 37°C for 5 or 24 hours followed by microscopic examination and photographed. Capillary formation reached the highest level when both groups of cells were mixed in a 1:1 ratio. (D) GFP- and miR-328-transfected cells with or without mix (GFP/miR-328 in 1:1 ratio) were cultured in Matrigel in the presence or absence of 150 μg/ml hyaluronan at 37°C for 24 hours. Addition of hyaluronan promoted the formation of capillary structure.

## Supporting information

S1 File Available data underlying Fig 2C 5 h 1/4 panel. (JPG)

S2 File Available data underlying Fig 2C 5 h 1/2 panel. (JPG)

S3 File Available data underlying Fig 2C 5 h 1/1 panel. (JPG)

S4 File Available data underlying Fig 2C 5 h 2/1 panel. (JPG)

S5 File Available data underlying Fig 2C 5 h 4/1 panel. (JPG)

S6 File Available data underlying Fig 2C 24 h 1/4 panel. (JPG)

S7 File Available data underlying Fig 2C 24 h 1/2 panel. (JPG)

S8 File Available data underlying Fig 2C 24 h 1/1 panel. (JPG)

S9 File Available data underlying Fig 2C 24 h 2/1 panel. (JPG)

S10 File Available data underlying Fig 2C 24 h 4/1 panel. (JPG)
