# Supplementary material for: MicroRNA miR-328 Regulates Zonation Morphogenesis by Targeting CD44 Expression
Source: PLoS One. 2008 Jun 18;3(6):e2420. doi: 10.1371/journal.pone.0002420 (PMC2409976; doi:10.1371/journal.pone.0002420)
Supplement: Table S1 — (0.04 MB DOC) [file pone.0002420.s001.doc]

**Table S1. Primers Used in This Study**

| **primer** | **sequence** |
| --- | --- |
| RT-328-2 | 5’ggagatgaattcttatgatattacgcataacaatacatcaccgtaagcacggaagggcag |
| PCR-328-2 | 5’aaaaaatttccaaggcctctggccctctct |
| PCR-miRNA-3 | 5’aattctaaattatctaatataggagatgaattcttatgatattac |
| huCD44-si868p1 | 5’tacgtacgtcccagatcttaactattgttaaccgtgatggcatcaagacgccatcacggttaacaatagttagactgccgaccagcagagcagttg |
| huCD44-si1115p2 | 5’gggctaaagcttaaaaaatcatagtataacgcttcagccagagaaagggctgaagcgttatactatgactgctctgctggtcggca |
| huCD443*E*1917FSacI | 5’cccggggagctcgaattccacctacaccattatcttggaaag |
| huCD443*E*2884RMluI | 5’gggcccacgcgtagaggctcaagctcctcagcttcc |
| huCD443*E*2784RMluI | 5’gggcccacgcgtagtgctgccctccttggt |
| huCD443*E*2697RMluI | 5’gggcccacgcgttctccttaccattctcag |
| huCD443*E*2680RMluI | 5’gggcccacgcgtgctcttcctgatacattg |
| miR-328N | 5’agatcttggagtgggagggca |
| miR-328C | 5’caggggacggaagggcagaga |
| hu-GAPDH-131F | 5’actctggtaaagtgcatattgttg |
| hu-GAPDH-380R | 5’ttctccatggtggtcaagtcgcca |
| chver10051-SpeI | 5’gggcccactagtaatggagccacatgtatagat |
| chver10350-SacI | 5’gggcccgagctcgaaatcacgctcaaacatctt |
| huCD44-2833R-mu | 5’acctttggacagtgtcaaaaaaaaaaaacaaaaaacaaaaaacaaaaacagtgctcgggagcttggtcttccatgagatttggctgag |
| huCD44-2884R | 5’gggcccacgcgtagaggctgaagctcctcagcttccaactctgattccaggacaggatggaaaacctttggacagtgtcaa |
| huCD44-2884R-mu | 5’gggcccacgcgtagaggctgaacgtggagagcttccaactctgattccaggacaggatggaaaacctttggacagtgtcaa |
