# Supplementary material for: MicroRNA miR-328 Regulates Zonation Morphogenesis by Targeting CD44 Expression
Source: PLoS One. 2008 Jun 18;3(6):e2420. doi: 10.1371/journal.pone.0002420 (PMC2409976; doi:10.1371/journal.pone.0002420)
Supplement: Figure S1 — A, Diagram of the procedure of RT-PCR of mature microRNA miR-328. The mRNA was isolated with mirVana miRNA Isolation Kit (Ambion, Austin, TX). RT-PCR was performed using Superscript II Reverse Transcriptase (Invitrogen). PCR was carried out at the temperature of 94°C, 56°C, and 72°C for 25 cycles. B, RT-PCR of mature miR-328, miR-378, miR-17-3p, and miR-17-5p using RNA prepared from A431 cells stably transfected with miR-328 and a control vector, confirming that processing of other microRNAs was not affected by miR-328 transfection. (0.12 MB PPT) [file pone.0002420.s002.ppt]

## Slide 1
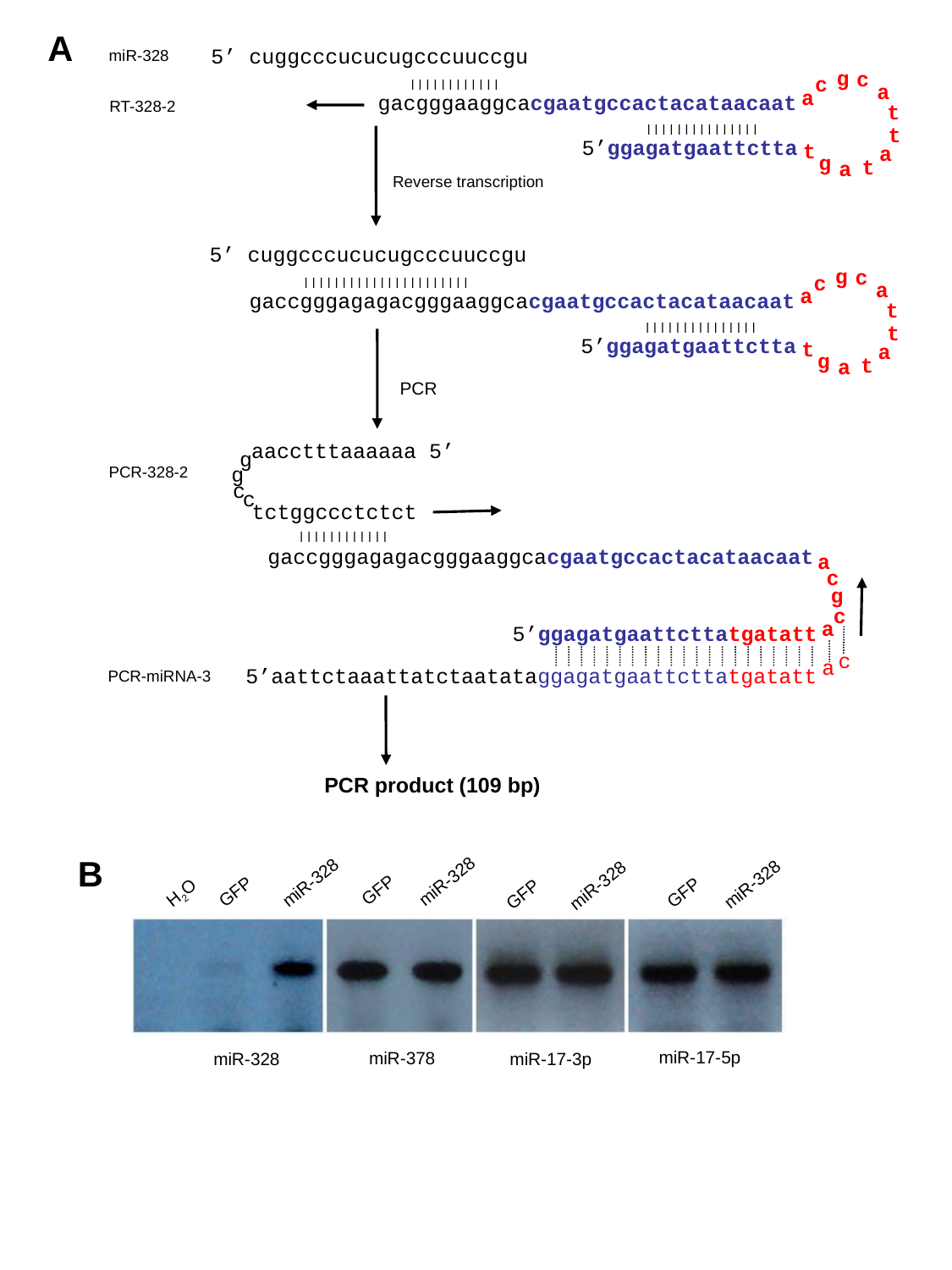

5’ cuggcccucucugcccuuccgu
A
miR-328
g
c
c
a
a
t
t
t
a
g
t
a

gacgggaaggcacgaatgccactacataacaat
RT-328-2

5’ggagatgaattctta
Reverse transcription
5’ cuggcccucucugcccuuccgu
g
c
c
a
a
t
t
t
a
g
t
a

gaccgggagagacgggaaggcacgaatgccactacataacaat

5’ggagatgaattctta
PCR
aacctttaaaaaa 5’
g
g
PCR-328-2
c
c
tctggccctctct

gaccgggagagacgggaaggcacgaatgccactacataacaat
a
c
g
c
a
5’ggagatgaattcttatgatatt
c
a
5’aattctaaattatctaatataggagatgaattcttatgatatt
PCR-miRNA-3
PCR product (109 bp)
B
miR-328
miR-328
miR-328
miR-328
GFP
GFP
H2O
GFP
GFP
miR-17-5p
miR-378
miR-328
miR-17-3p
