# Supplementary figures and images for: MicroRNA miR-328 Regulates Zonation Morphogenesis by Targeting CD44 Expression
Source: PLoS One. 2008 Jun 18;3(6):e2420. doi: 10.1371/journal.pone.0002420 (PMC2409976; doi:10.1371/journal.pone.0002420)

## Slide 1
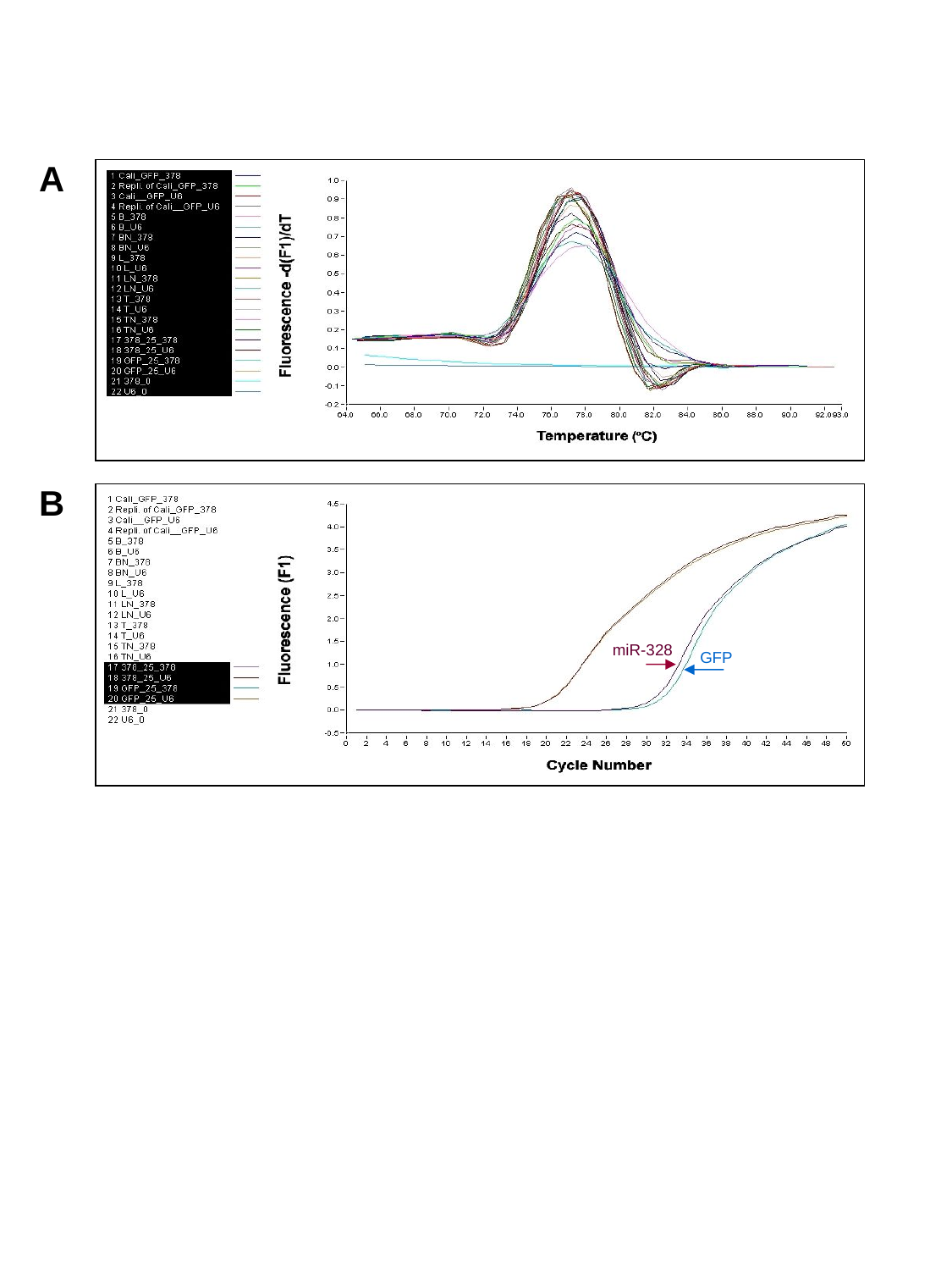

A
B
miR-328
GFP

Supplement: Figure S2 — A, The melting curves in real-time PCR experiments reveal undetectable contamination, mispriming, and primer-dimer artifacts of all samples indicated on the left of the curves. B, Real-time PCR curves of mature miR-328 from RNAs isolated from miR-328- and GFP vector-transfected cells. Real-time PCR was carried out according to the manufacturer's instructions (Qiagen, miScript Reverse Transcription Kit, cat #218060; miScript Primer Assay, cat #218411; miScript SYBR Green PCR Kit, cat #218073). (0.23 MB PPT) [file pone.0002420.s003.ppt]

## Slide 1
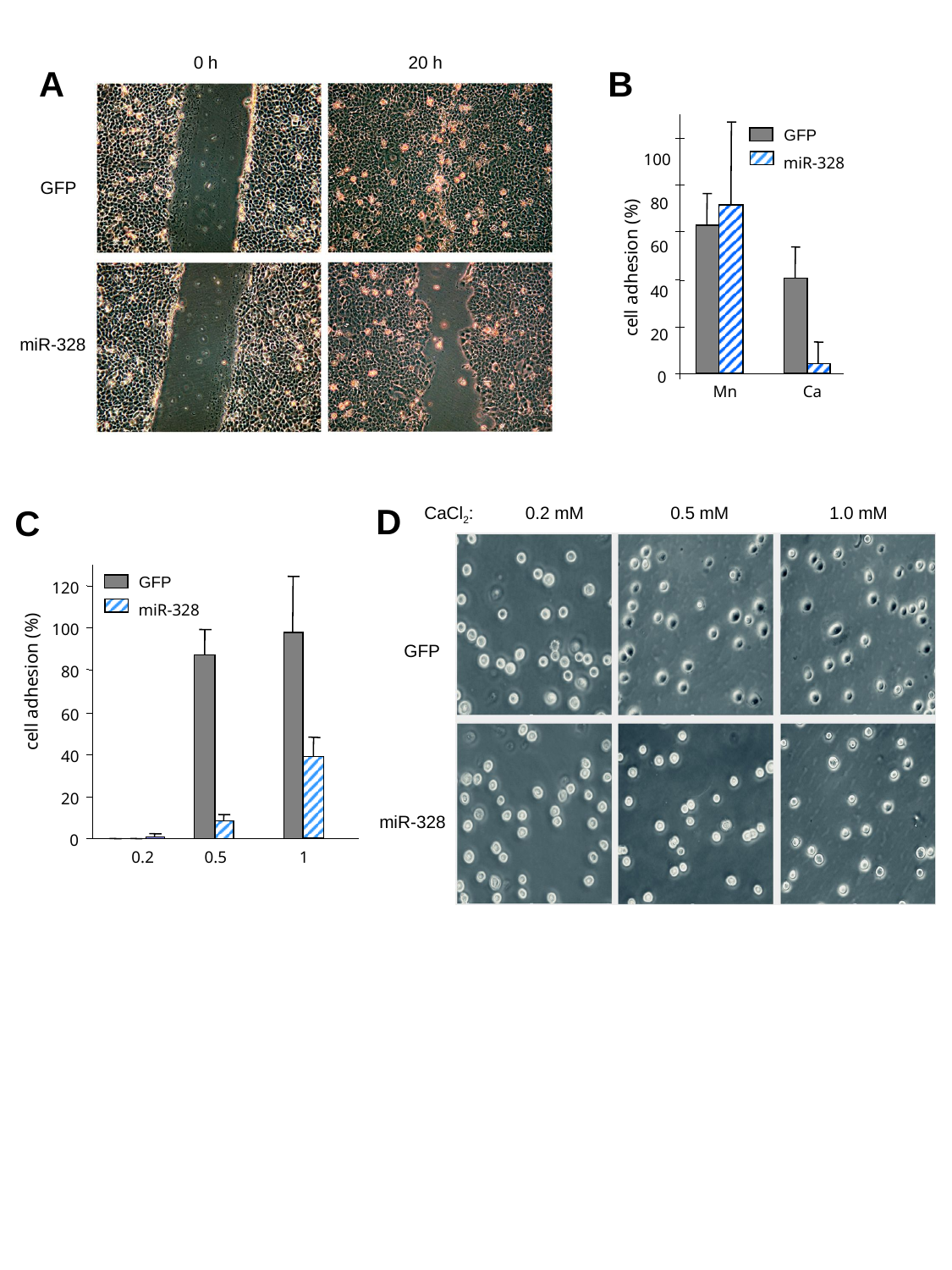

0 h
20 h
GFP
miR-328
A
B
GFP
miR-328
100
80
60
cell adhesion (%)
40
20
0
Ca
Mn
D
C
CaCl2:
0.2 mM
0.5 mM
1.0 mM
GFP
120
miR-328
100
GFP
80
cell adhesion (%)
60
40
20
miR-328
0
1
0.2
0.5

Supplement: Figure S3 — A, GFP- and miR-328-transfected cells were cultured on tissue culture plates to confluence. The cultures were wounded by a micro-tip. Migration of cells to the wounding areas was examined under a light microscope and photographed. [Cell migration assay: Wound healing experiments were used to examine cell migration. Cells (3×10^6^) were seeded on 3.5 cm culture dishes in DMEM supplemented with 10% FBS. Twenty-four hours after cell inoculation, sub-confluent monolayers were wounded linearly by scraping with p1000 pipette tips, washed to remove cell debris and refilled with fresh media. The images were captured at the beginning and 15 hours later with a phase-contrast microscope.] B, GFP- and miR-328-transfected cells were incubated in Petri dishes in the presence of HBSS with manganese (Mn) or calcium (Ca) for 6 hours. C, GFP- and miR-328-transfected cells were incubated on Petri dishes in the presence of 0.2, 0.5, and 1.0 mM CaCl2 for 6 hours. Cell adhesion was analyzed by counting the cells that adhered to plates. miR-328-transfected cells exhibited lower rates of adhesion than the GFP-transfected cells. Error bars, SD (n = 8). D, typical micrographs of cell attachment are shown. (2.64 MB PPT) [file pone.0002420.s004.ppt]

## Slide 1
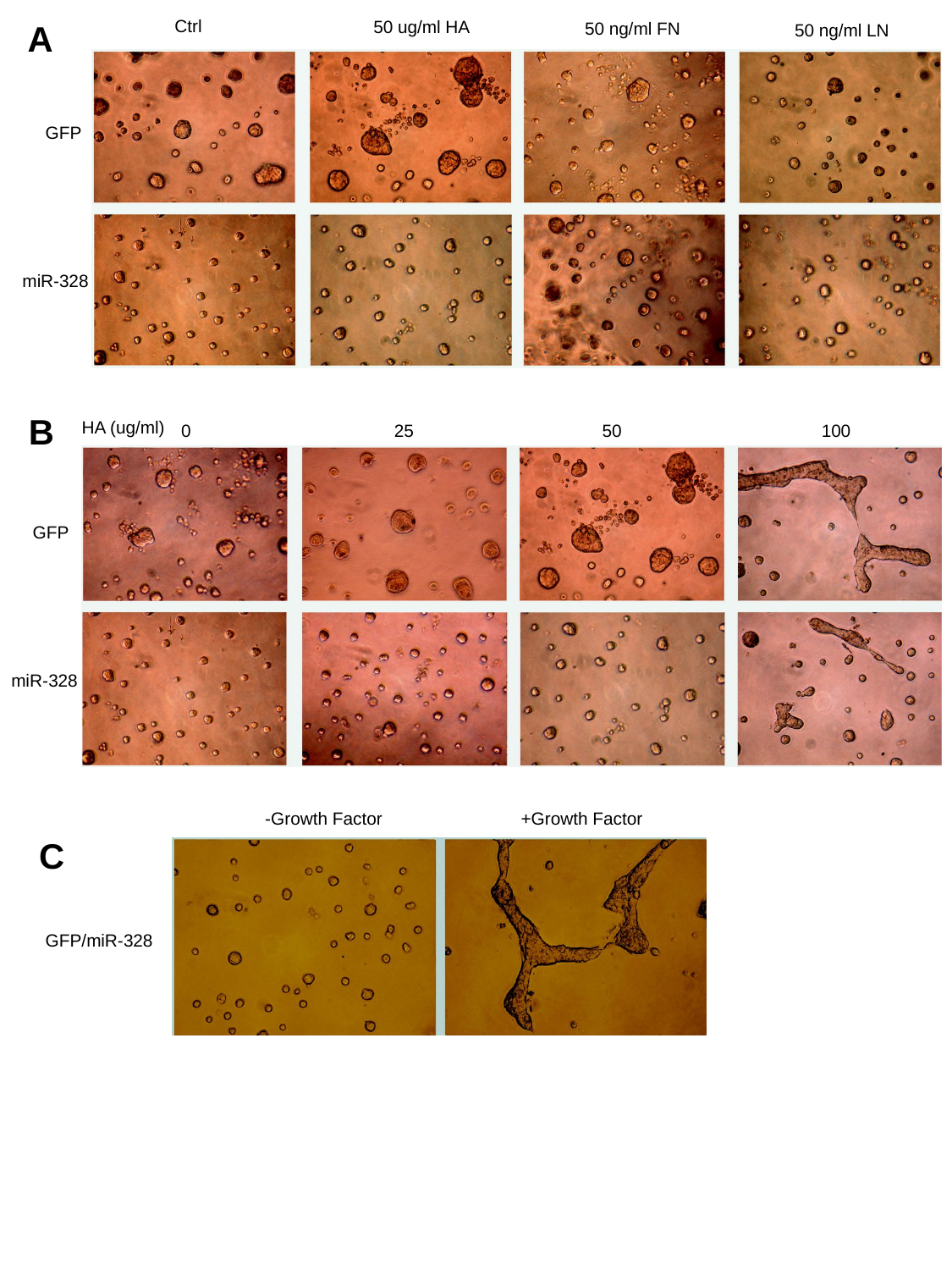

Ctrl
50 ug/ml HA
A
50 ng/ml FN
50 ng/ml LN
GFP
miR-328
B
HA (ug/ml)
0
25
50
100
GFP
miR-328
-Growth Factor
+Growth Factor
C
GFP/miR-328

Supplement: Figure S5 — A, GFP- and miR-328-transfected cells were incubated in Matrigel without (Ctrl) or with hyaluronan (HA, 50 ng/ml), fibronectin (FN, 50 ng/ml), and laminin (LN, 50 ng/ml). The cultures were maintained at 37°C for 24 hours followed by microscopic examination and photographed. Reduction of cell aggregation was observed in the miR-328-transfected cells. B, The cells were also incubated in Matrigel with the addition of 0, 25, 50, and 100 mg/ml hyaluronan for 24 hours. Addition of hyaluronan promoted tube-like structure formation. C, GFP- and miR-328-transfected cells were mixed in a 1:1 ratio. The mixed cells were incubated in Matrigel with or without growth factors (GF) at 37°C for 24 hours followed by microscopic examination and photographed. Presence of growth factors promoted the formation of tube-like structures. μ (7.07 MB PPT) [file pone.0002420.s006.ppt]

## Slide 1
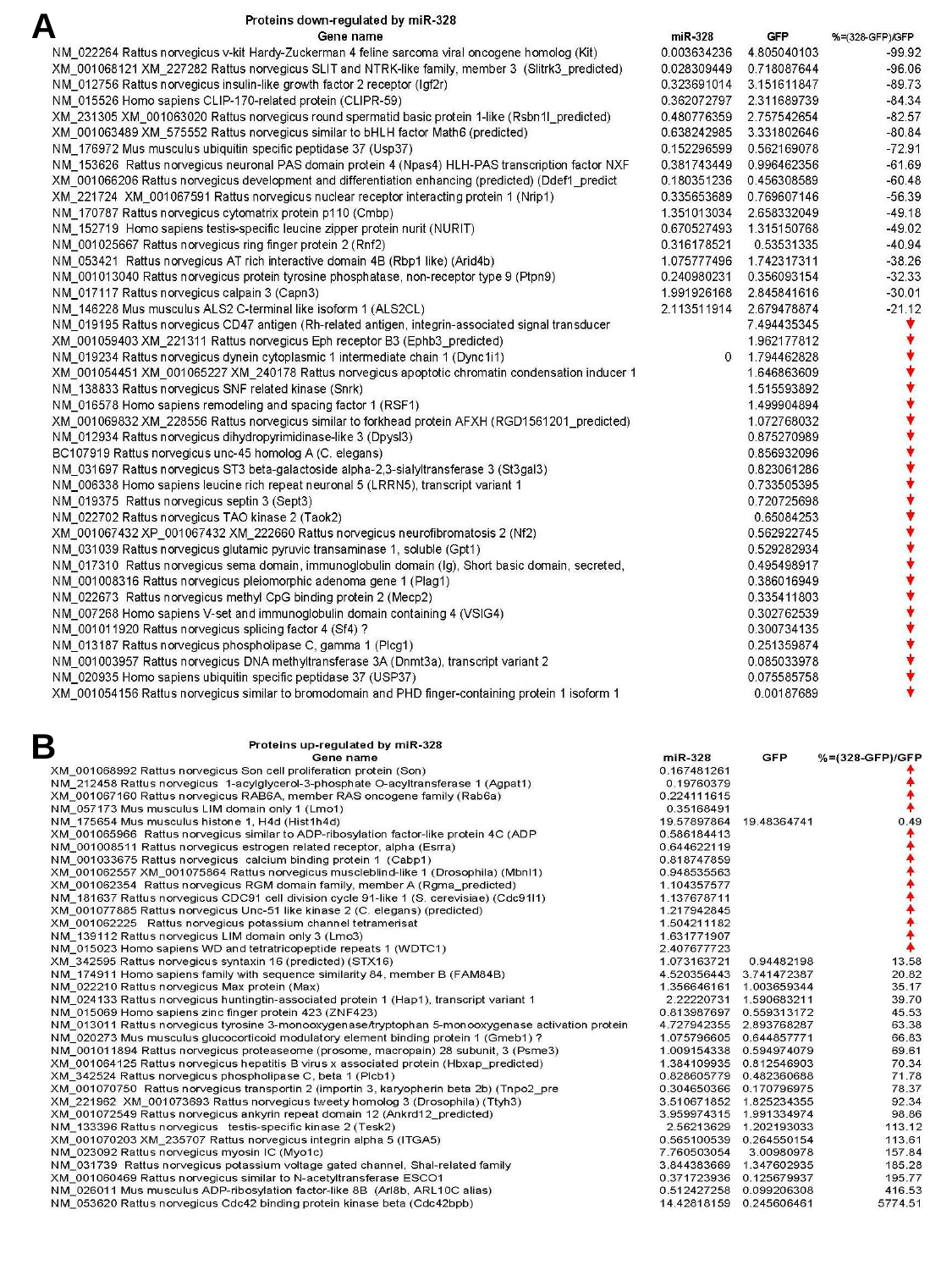

A
B

Supplement: Figure S7 — Proteomic results. GFP- and miR-328-transfected cells were cultured on tissue culture plates to confluence. Cell lysate was prepared and subjected to proteomic analysis. A number of proteins were detected to be down-regulated (A) and up-regulated (B) by miR-328-transfection. (2.30 MB PPT) [file pone.0002420.s008.ppt]
