# Supplementary material for: MicroRNA miR-328 Regulates Zonation Morphogenesis by Targeting CD44 Expression
Source: PLoS One. 2008 Jun 18;3(6):e2420. doi: 10.1371/journal.pone.0002420 (PMC2409976; doi:10.1371/journal.pone.0002420)
Supplement: Figure S4 — A, Expression of miR-328 reduces cell adhesion. GFP- and miR-328-transfected cells were incubated in Petri dishes precoated without (Ctrl) or with fibronectin (FN, 50 mg/ml), hyaluronan (HA, 5 mg/ml), and laminin (LN, 50 mg/ml). The cultures were maintained at 37°C for 3 hours in culture medium followed by microscopic examination. Adherent cells were counted. Reduction of cell adhesion was observed in the miR-328-transfected cells. B, FP- and miR-328-transfected cells were incubated in Matrigel with or without growth factors. C, cell number was counted to determine rates of proliferation. [Proliferation assay: 2×10^5^ /well of vector- and miR-328-transfected A431 cells were seeded to 6-well plastic tissue culture plates in DMEM containing 10% FBS, and incubated for different time periods. The cultures were trypsinised and concentrated to a volume of 100 nl for counting by hemocytometer (n = 3±S.D.).] μ (1.12 MB PPT) [file pone.0002420.s005.ppt]

## Slide 1
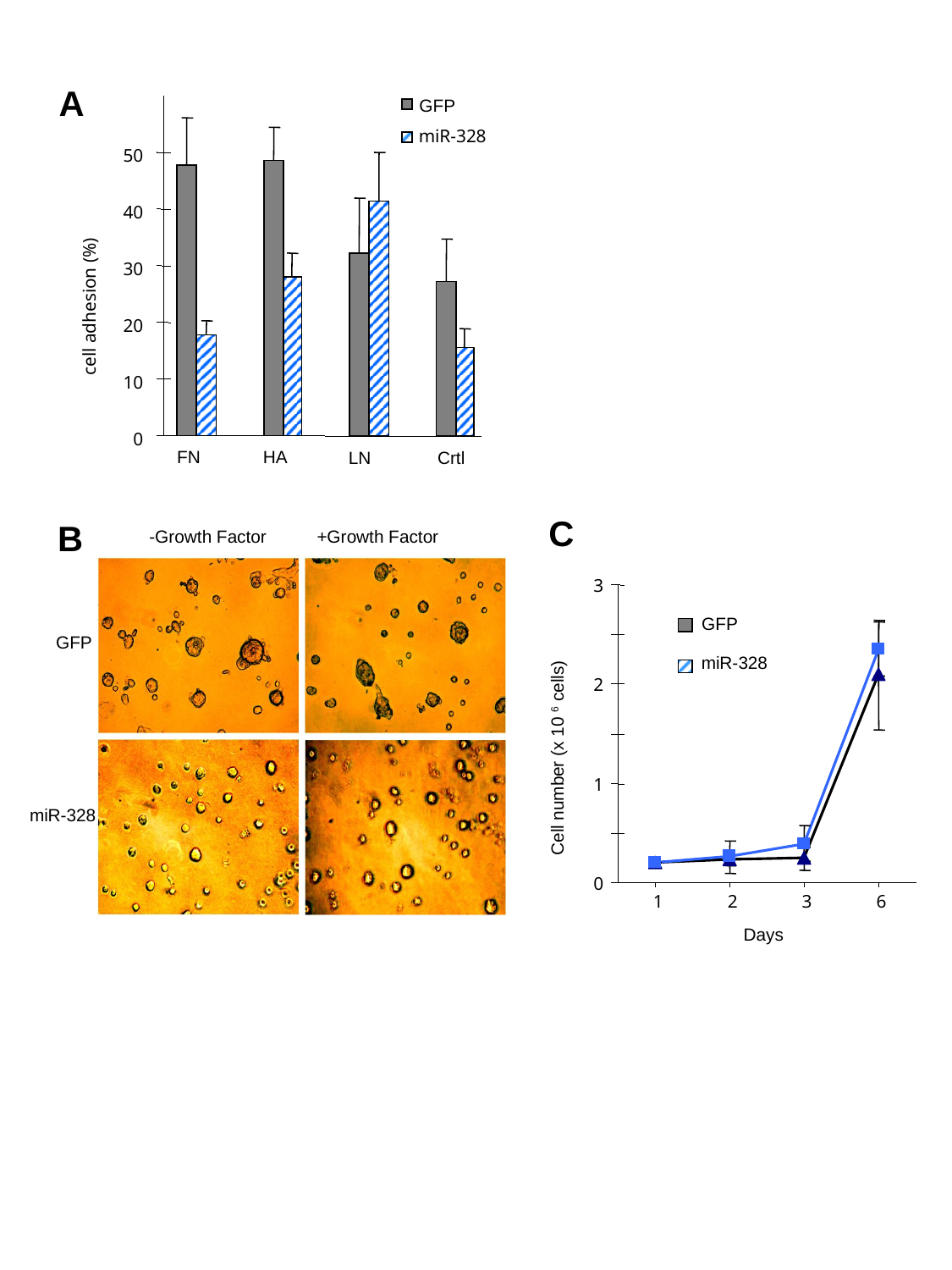

A
GFP
miR-328
50
40
30
cell adhesion (%)
20
10
0
FN
HA
Crtl
LN
C
B
-Growth Factor
+Growth Factor
3
GFP
miR-328
2
Cell number (x 10 6 cells)
1
0
1
2
3
6
Days
GFP
miR-328
