# Supplementary material for: MicroRNA miR-328 Regulates Zonation Morphogenesis by Targeting CD44 Expression
Source: PLoS One. 2008 Jun 18;3(6):e2420. doi: 10.1371/journal.pone.0002420 (PMC2409976; doi:10.1371/journal.pone.0002420)
Supplement: Figure S6 — A, GFP- and miR-328-transfected cells were cultured on tissue culture plates to confluence. Cell lysate was prepared and subjected to Western blotting probing with antibodies against different adhesion molecules as indicated. Little difference was detected. B, GFP-, miR-328-, and anti-miR-328 (antisense against miR-328)-transfected cells were cultured on tissue culture plates to confluence. Cell lysate was prepared and subjected to Western blotting probing with antibodies against CD44 or actin. C, RNA samples were also prepared for RT-PCR amplifying pre-miR-328 and mature miR-328. (0.99 MB PPT) [file pone.0002420.s007.ppt]

## Slide 1
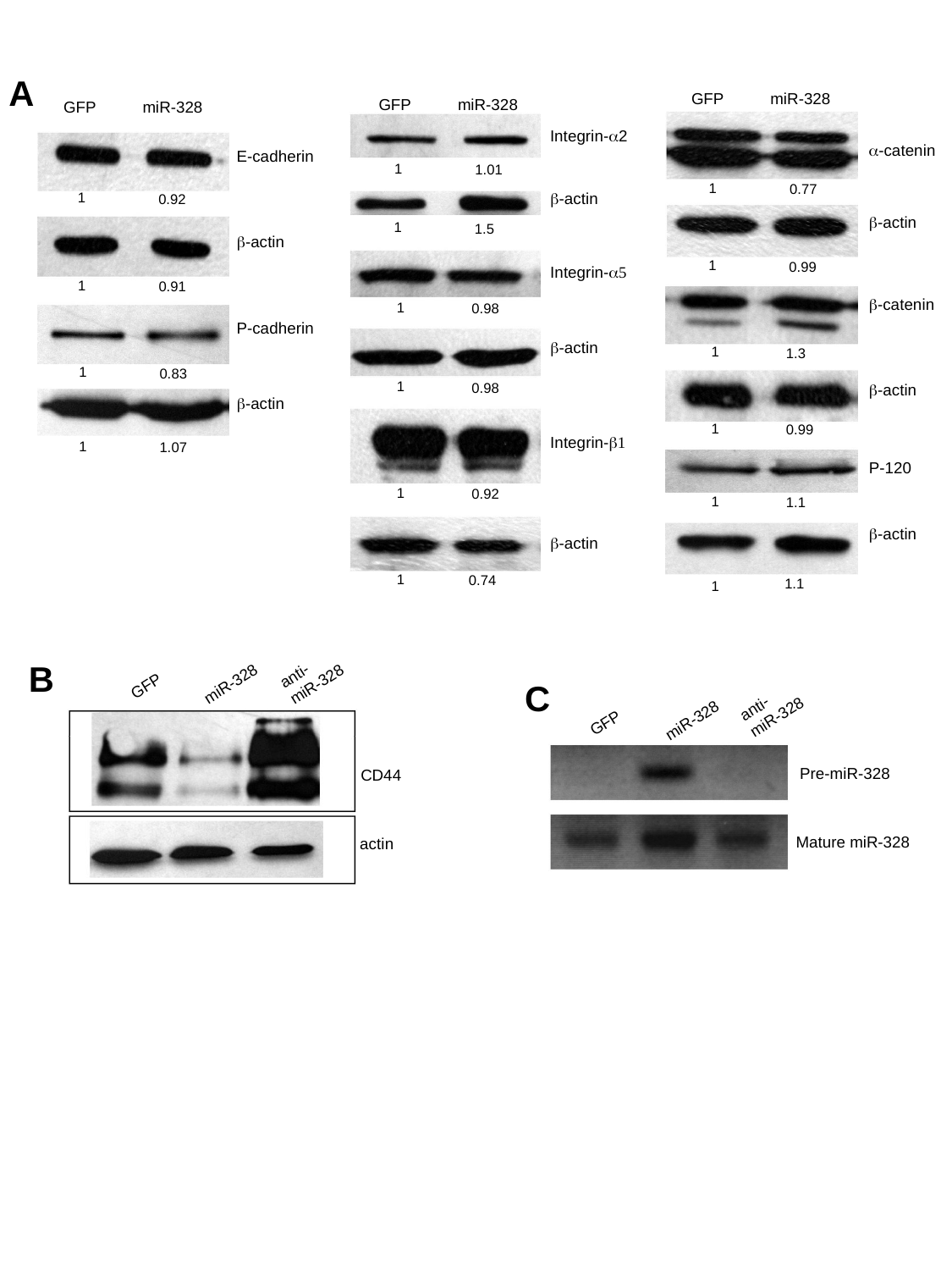

A
GFP
miR-328
GFP
miR-328
GFP
miR-328
Integrin-α2
α-catenin
E-cadherin
1
1.01
1
0.77
β-actin
1
0.92
β-actin
1
1.5
β-actin
1
0.99
Integrin-
1
0.91
β-catenin
1
0.98
P-cadherin
β-actin
1
1.3
1
0.83
1
0.98
β-actin
β-actin
1
0.99
Integrin-
1
1.07
P-120
1
0.92
1
1.1
β-actin
β-actin
1
0.74
1.1
1
anti-
miR-328
B
miR-328
GFP
C
anti-
miR-328
miR-328
GFP
Pre-miR-328
CD44
Mature miR-328
actin
