# Supplementary material for: MicroRNA miR-328 Regulates Zonation Morphogenesis by Targeting CD44 Expression
Source: PLoS One. 2008 Jun 18;3(6):e2420. doi: 10.1371/journal.pone.0002420 (PMC2409976; doi:10.1371/journal.pone.0002420)
Supplement: Figure S8 — Luciferase report constructs for CD44. Fragments of CD44 3'UTR were inserted into the luciferase report vector pMiR-Report producing a constructs named Luc-CD44a, Luc-CD44b, Luc-CD44c, and Luc-CD44d . The potential mir-328 target sequences were labelled in blue. A control sequence (Ctrl) was obtained from the G3 domain of chicken versican. (0.04 MB PPT) [file pone.0002420.s009.ppt]

## Slide 1
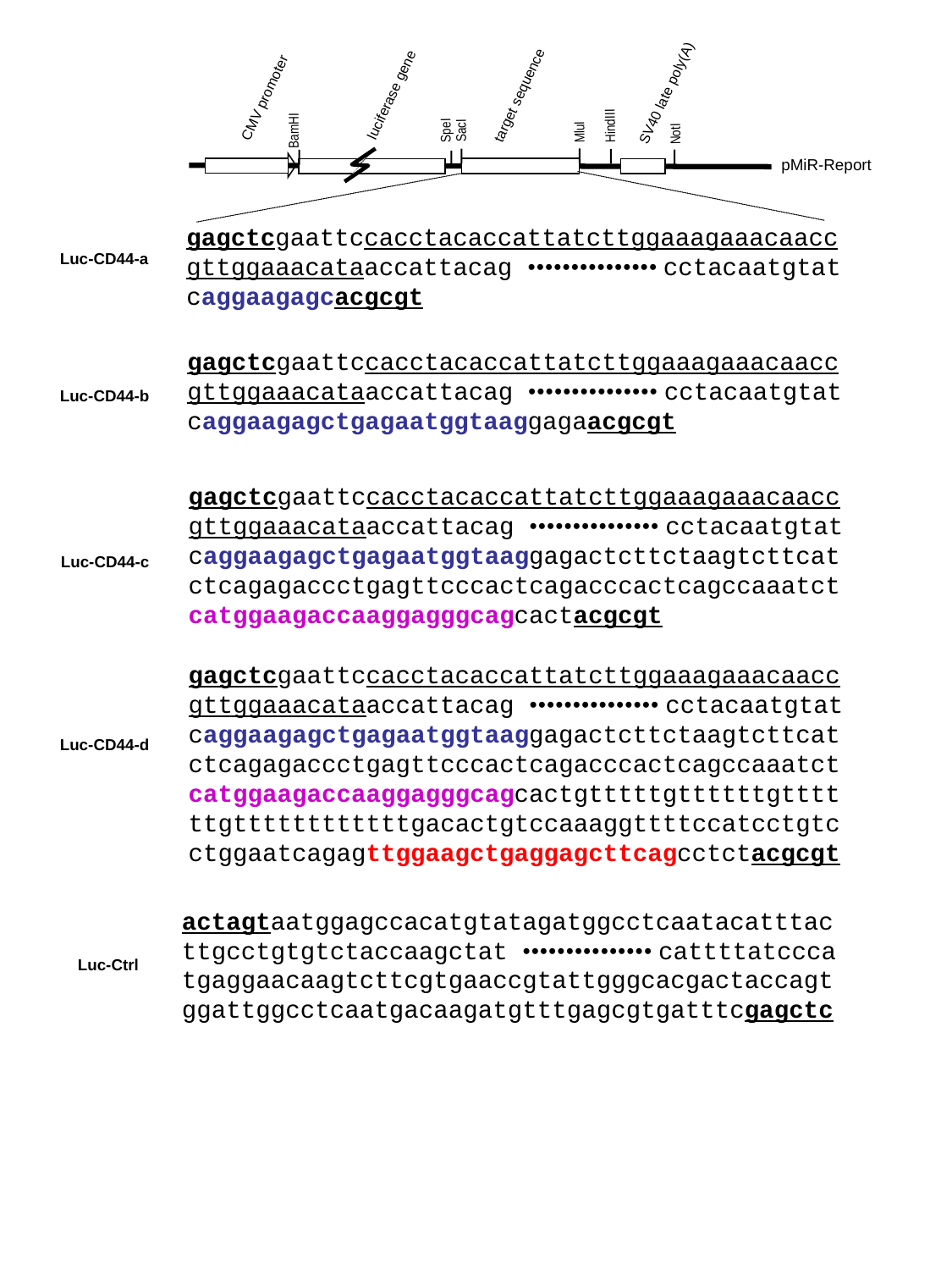

SV40 late poly(A)
luciferase gene
target sequence
CMV promoter
HindIII
SacI
SpeI
BamHI
MluI
NotI
pMiR-Report
gagctcgaattccacctacaccattatcttggaaagaaacaaccgttggaaacataaccattacag ••••••••••••••• cctacaatgtat
caggaagagcacgcgt
Luc-CD44-a
gagctcgaattccacctacaccattatcttggaaagaaacaaccgttggaaacataaccattacag ••••••••••••••• cctacaatgtat
caggaagagctgagaatggtaaggagaacgcgt
Luc-CD44-b
gagctcgaattccacctacaccattatcttggaaagaaacaaccgttggaaacataaccattacag ••••••••••••••• cctacaatgtat
caggaagagctgagaatggtaaggagactcttctaagtcttcatctcagagaccctgagttcccactcagacccactcagccaaatctcatggaagaccaaggagggcagcactacgcgt
Luc-CD44-c
gagctcgaattccacctacaccattatcttggaaagaaacaaccgttggaaacataaccattacag ••••••••••••••• cctacaatgtat
caggaagagctgagaatggtaaggagactcttctaagtcttcatctcagagaccctgagttcccactcagacccactcagccaaatctcatggaagaccaaggagggcagcactgtttttgttttttgttttttgttttttttttttgacactgtccaaaggttttccatcctgtcctggaatcagagttggaagctgaggagcttcagcctctacgcgt
Luc-CD44-d
actagtaatggagccacatgtatagatggcctcaatacatttacttgcctgtgtctaccaagctat ••••••••••••••• cattttatccca
tgaggaacaagtcttcgtgaaccgtattgggcacgactaccagtggattggcctcaatgacaagatgtttgagcgtgatttcgagctc
Luc-Ctrl
