# Supplementary material for: MicroRNA miR-328 Regulates Zonation Morphogenesis by Targeting CD44 Expression
Source: PLoS One. 2008 Jun 18;3(6):e2420. doi: 10.1371/journal.pone.0002420 (PMC2409976; doi:10.1371/journal.pone.0002420)
Supplement: Figure S9 — A, GFP- and miR-328-transfected cells were cultured in the presence or absence of anti-CD44 antibody or hyaluronidase at 37°C for 48 hours followed by viability assays using an MTT assay kit. (Toxicity assay: Cells were seeded at a concentration of 2×10^3^ cells/well in 96-well plate without or with anti-CD44 antibody or hyaluronidase for 48 hours. MTT assay was used for detecting cell viability.) B, siRNA construct for CD44 (left). siRNA-mediated silencing of CD44 was examined by Western blot probed with anti-CD44 antibody (right). C, Tumor tissue stained with CD44. Tumors formed by astrocytoma cells U87 were subjected to immunohistochemistry with anti-CD44 antibody. CD44-negative layers as indicated by the brackets were detected between the tumor tissue and the stroma tissue. (1.13 MB PPT) [file pone.0002420.s010.ppt]

## Slide 1
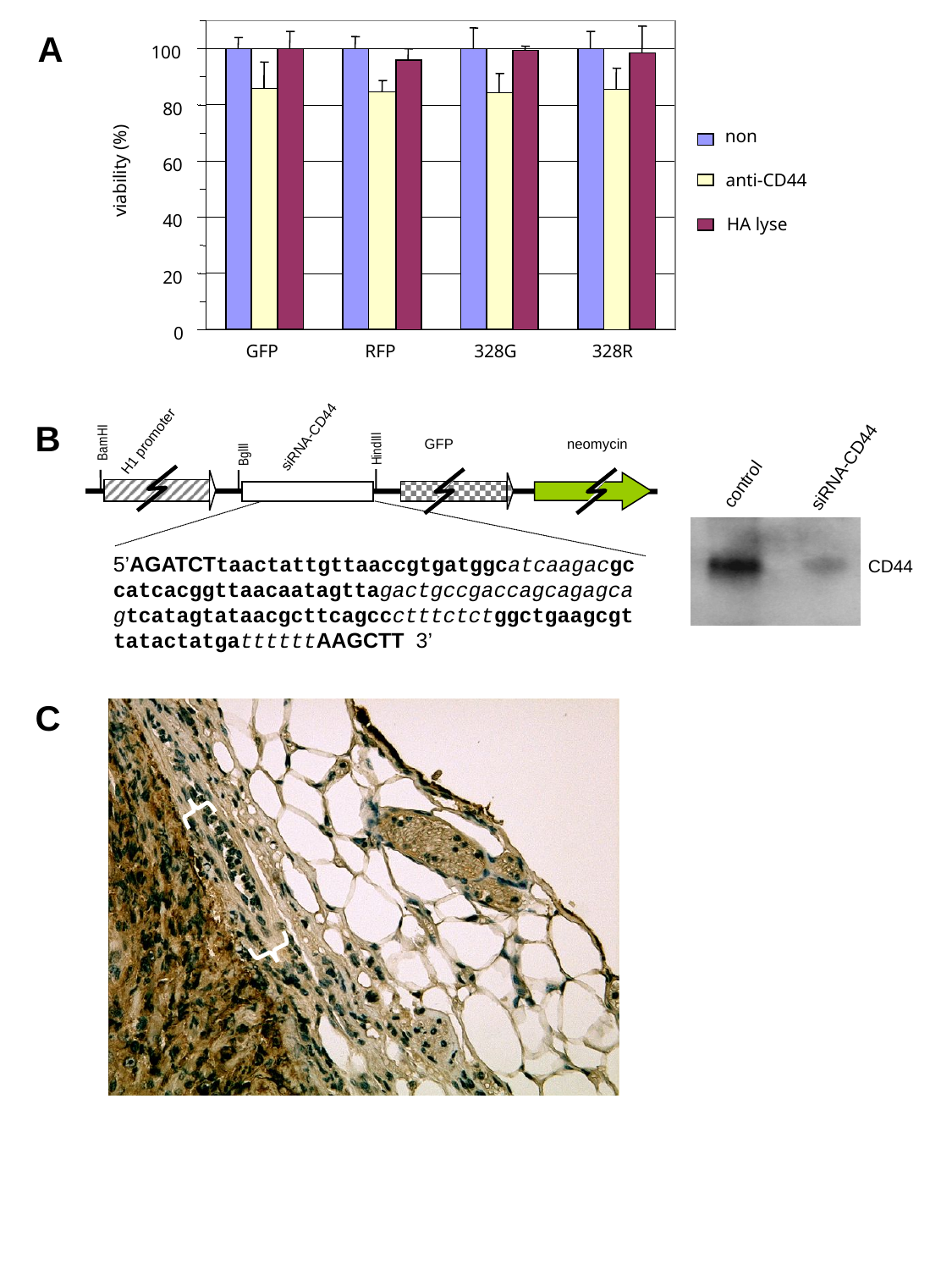

A
100
80
non
60
viability (%)
anti-CD44
40
HA lyse
20
0
GFP
RFP
328G
328R
siRNA-CD44
H1 promoter
BamHI
neomycin
GFP
HindIII
BglII
B
siRNA-CD44
control
5’AGATCTtaactattgttaaccgtgatggcatcaagacgccatcacggttaacaatagttagactgccgaccagcagagcagtcatagtataacgcttcagccctttctctggctgaagcgttatactatgattttttAAGCTT 3’
CD44
C
